# Independent relationship between sleep apnea-specific hypoxic burden and glucolipid metabolism disorder: a cross-sectional study

**DOI:** 10.1186/s12931-024-02846-7

**Published:** 2024-05-18

**Authors:** Chenyang Li, Yu Peng, Xiaoyue Zhu, Yupu Liu, Jianyin Zou, Huaming Zhu, Xinyi Li, Hongliang Yi, Jian Guan, Xu Zhang, Huajun Xu, Shankai Yin

**Affiliations:** 1 Department of Otolaryngology-Head and Neck Surgery & Center of Sleep Medicine, Shanghai Jiao Tong University School of Medicine Affiliated Sixth People’s Hospital, Shanghai, China; 2 Shanghai Key Laboratory of Sleep Disordered Breathing, Shanghai, China; 3 School of Biomedical Engineering, Shenzhen University Medical School, Shenzhen, China

**Keywords:** Obstructive sleep apnea hypopnea syndrome, Sleep apnea-specific hypoxic burden, Abnormal glucose and lipid metabolism.

## Abstract

**Objectives:**

Obstructive sleep apnea (OSA) is associated with abnormal glucose and lipid metabolism. However, whether there is an independent association between Sleep Apnea-Specific Hypoxic Burden (SASHB) and glycolipid metabolism disorders in patients with OSA is unknown.

**Methods:**

We enrolled 2,173 participants with suspected OSA from January 2019 to July 2023 in this study. Polysomnographic variables, biochemical indicators, and physical measurements were collected from each participant. Multiple linear regression analyses were used to evaluate independent associations between SASHB, AHI, CT90 and glucose as well as lipid profile. Furthermore, logistic regressions were used to determine the odds ratios (ORs) for abnormal glucose and lipid metabolism across various SASHB, AHI, CT90 quartiles.

**Results:**

The SASHB was independently associated with fasting blood glucose (FBG) (β = 0.058, *P* = 0.016), fasting insulin (FIN) (β = 0.073, *P* < 0.001), homeostasis model assessment of insulin resistance (HOMA-IR) (β = 0.058, *P* = 0.011), total cholesterol (TC) (β = 0.100, *P* < 0.001), total triglycerides (TG) (β = 0.063, *P* = 0.011), low-density lipoprotein cholesterol (LDL-C) (β = 0.075, *P* = 0.003), apolipoprotein A-I (apoA-I) (β = 0.051, *P* = 0.049), apolipoprotein B (apoB) (β = 0.136, *P* < 0.001), apolipoprotein E (apoE) (β = 0.088, *P* < 0.001) after adjustments for confounding factors. Furthermore, the ORs for hyperinsulinemia across the higher SASHB quartiles were 1.527, 1.545, and 2.024 respectively, compared with the lowest quartile (*P* < 0.001 for a linear trend); the ORs for hyper-total cholesterolemia across the higher SASHB quartiles were 1.762, 1.998, and 2.708, compared with the lowest quartile (*P* < 0.001 for a linear trend) and the ORs for hyper-LDL cholesterolemia across the higher SASHB quartiles were 1.663, 1.695, and 2.316, compared with the lowest quartile (*P* < 0.001 for a linear trend). Notably, the ORs for hyper-triglyceridemia{1.471, 1.773, 2.099} and abnormal HOMA-IR{1.510, 1.492, 1.937} maintained a consistent trend across the SASHB quartiles.

**Conclusions:**

We found SASHB was independently associated with hyperinsulinemia, abnormal HOMA-IR, hyper-total cholesterolemia, hyper-triglyceridemia and hyper-LDL cholesterolemia in Chinese Han population. Further prospective studies are needed to confirm that SASHB can be used as a predictor of abnormal glycolipid metabolism disorders in patients with OSA.

**Trial registration:**

ChiCTR1900025714 {http://www.chictr.org.cn/}; Prospectively registered on 6 September 2019; China.

**Supplementary Information:**

The online version contains supplementary material available at 10.1186/s12931-024-02846-7.

## Introduction

Obstructive sleep apnea (OSA) is a highly prevalent sleep disordered breathing, with estimated prevalence rates of 17% in females and 34% in males within the general population, and increasingly with age and obesity [1, 2]. OSA is characterized by repeated intermittent hypoxia (IH), frequent arousals, and daytime drowsiness. A significant association between OSA and the risk of metabolic syndrome was recently established and attracted considerable attention [3, 4]. Dyslipidemia and abnormal glucose metabolism emerge as two primary components of metabolic disorders, and both of them are commonly intertwined with clinical outcomes associated with OSA [5, 6]. Previous studies have confirmed that OSA is associated with dyslipidemia, abnormal glucose metabolism and such an association is mainly attributable to nocturnal IH and sleep fragmentation (SF) [7–10]. Previous rodent studies have suggested that IH can impair pancreatic islet beta-cell function, subsequently increasing fasting glucose levels and generating insulin resistance [11, 12], thereby further exacerbating the development of diabetes [13, 14].

In our previous study [15], we found Sleep Apnea-Specific Hypoxic Burden (SASHB), a new pulse oximetry (SpO_2_)-related index and defined as the sum of the areas under the baseline SpO_2_ curves corresponding to respiratory events, has shown promise in identifying people at risk of OSA in Chinese Han population. In addition to quantifying the frequency of respiratory events, SASHB captures the depth and duration of hypoxemia associated with OSA. Series of studies have shown that higher SASHB in OSA were associated with higher risks of cardiovascular mortality, major cardiovascular event rates [16], blood pressure [17], stroke [18], heart failure [19], and chronic kidney disease in the clinical setting [20]. However, whether SASHB was independently associated with abnormal glucose and lipid metabolism remains unknown.

In order to clearly address such an association, we performed such comprehensive cross-sectional study. Furthermore, we calculated adjusted odds ratios (ORs) for different abnormal glucose and lipid metabolism categories among OSA patients, stratified by varying levels of SASHB.

## Subjects and methods

### Study design and population

**Fig. 1:**
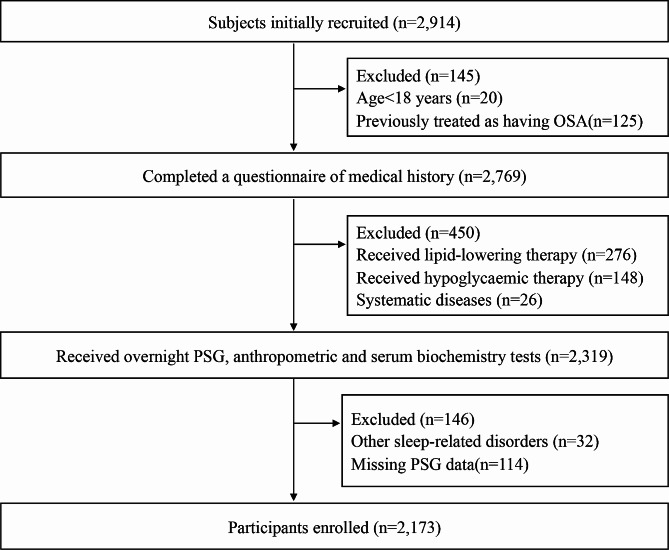
Screening flow chat of participants

We enrolled 2,914 subjects with suspected OSA who underwent overnight polysomnography (PSG) in the sleep laboratory of Shanghai Jiao Tong University of Medicine Affiliated Sixth People’s Hospital from January 2019 to July 2023. The exclusion criteria were as follows: (1) history of OSA treatment; (2) age < 18 years; (3) severe systemic disease such as heart, liver, lung, and renal failure; (4) other non-OSA sleep disorders; (5) severe psychiatric disorders or malignancy; (6) administration of glucose-lowering or lipid-lowering medications; and (7) missing clinical PSG data. Ultimately, a total of 2,173 subjects met the inclusion criteria for this study. We learned about their general health status including habits such as smoking, alcohol consumption, and medication use through a comprehensive questionnaire. The recruitment flow chart is shown in Fig. 1. This study was conducted in accordance with the Helsinki Declaration and was approved by the Ethics Review Committee of the Sixth People’s Hospital which is affiliated with the Medical College of Shanghai Jiaotong University (approval no. 2019-KY-050 [K]); the study was registered in the China Clinical Trials Registry (serial number ChiCTR1900025714). All participants gave written informed consent.

### Polysomnography and definitions

To obtain precise and objective sleep parameters, sleep was monitored in the sleep laboratory by overnight PSG (Alice-5, Alice-6; Respironics, Pittsburgh, Pennsylvania, USA). Bilateral electroencephalogram (EEG) channels (C3-M2 and C4-M1), bilateral electrooculogram (EOG), chin electromyogram (EMG), lowest oxygen saturation, chest and abdominal wall motion, airflow, and body position were recorded for all study participants. The scoring of respiratory events, oxygen desaturations adhered to the guidelines established by the American Academy of Sleep Medicine (AASM) in 2017 [21]. Scoring of micro-arousals followed the Rechtschaffen and Kales (R&K) rule [22]. A micro-arousal event was defined as an abrupt shift in EEG frequency, encompassing alpha, theta and/or frequencies > 16 Hz (excluding spindles) that lasted at least 3s, with at least 10s of stable sleep preceding the observed change. Additionally, scoring of arousal during rapid eye movement (REM) requires a concurrent increase in submental EMG lasting at least 1s. We operationally defined the microarousal index (MAI) as the tally of abrupt EEG frequency shifts, each lasting at least 3 s, per hour of recorded sleep. Moreover, the apnea hypopnea index (AHI) was quantified as the number of apnea and hypopnea events occurring per hour during the sleep period.

### Biochemical indicators

For each study participant, a fasting blood sample was collected from the antecubital vein on the morning following the PSG evaluation. Fasting blood glucose (FBG) and fasting insulin (FIN), and serum lipid profiles, which included total cholesterol (TC), total triglycerides (TG), high-density lipoprotein cholesterol (HDL-C), and low-density lipoprotein cholesterol (LDL-C), apolipoprotein A-I (apoA-I), apolipoprotein B (apoB), apolipoprotein E (apoE) were measured for each participant. FBG levels were quantified using the H-7600 autoanalyzer (Hitachi, Tokyo, Japan), while FIN levels were determined through immunoradiological assays. Calibration of the analyzer and quality control operations were routinely carried out. The homeostasis model assessment of insulin resistance (HOMA-IR) was calculated to quantify insulin resistance using FIN and FBG as follows: HOMA-IR = FIN (uIU/mL)×FBG (mmol/L)/22.5 [23]. We defined FBG of 6.1 mmol/L or greater as hyperglycemia, FIN of 12.2 uIU/mL or greater as hyperinsulinemia, and HOMA-IR of 2.5 or greater as insulin resistance [23–25].

Serum lipid profiles were assessed in the hospital laboratory utilizing standard procedures. According to the US National Cholesterol Education Program Adult Treatment Panel III (NCEPIII) [26] and the Joint Committee for Developing Chinese Guidelines on the Prevention and Treatment of Dyslipidemia in Adults (JCDCG) [27], dyslipidemia in terms of TC, LDL-c, HDL-c and TG, were defined as TC levels > 5.17mmol/L, LDL-c levels≥3.37mmol/L, HDL-c levels < 1.03 mmol/L, and TG levels ≥ 1.7 mmol/L, separately.

### Anthropometric measurements

All participants were instructed to wear light clothing and removed shoes. Height, weight, neck circumference (NC), waist circumference (WC), and hip circumference (HC) were measured with a meter ruler and weighing scale, respectively, following established procedures. Body Mass Index (BMI) was calculated as weight (kg)/height^2^(m^2^); the neck height ratio (NHR) = NC/height and the waist-hip ratio (WHR) = WC/HC were also calculated. Daytime blood pressure (BP) was measured after at least 5 min of rest in a seated position employing a mercury sphygmomanometer, following the American Society of Hypertension Guidelines, and the mean of three measurements was recorded for each participant. Hypertension was defined as a systolic BP ≥ 140mmHg, a diastolic BP ≥ 90mmHg, or current use of antihypertensive medication [28].

### The SASHB calculation flow

**Fig. 2:**
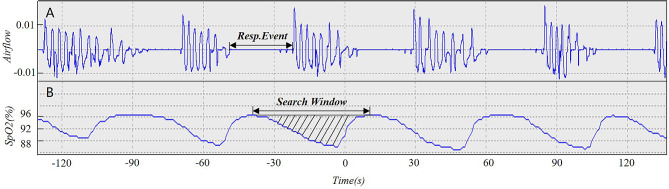
Calculation of SASHB for individual respiratory events corresponding to specific search window

The SASHB was determined by assessing the respiratory event-associated area under the desaturation curve commencing from a pre-event baseline. Our SASHB calculations are based on those of Dr. Azarbarzin’s research team, but the methods are not identical, differing principally in the definition of the pre-event SpO_2_ baseline level [15]. For each apnea or hypopnea event, the Azarbarzin team defined the pre-event baseline saturation as the maximum SpO_2_ during the 100 s prior to the end of the event; in our study, the maximum value at the start point of the SpO_2_ trend evident in the search window at the time of an apnea or hypopnea event served as the SpO_2_ baseline level. The search window used to detect respiratory events is shown in Fig. 2.

The specific SASHB calculation process was: First, take the maximum value of the starting point of SpO_2_ trend in the search window for each respiratory event as the SpO_2_ baseline level; Second, calculate${S}_{i}$, thus the area of the similar triangle for which the SpO_2_ level serves as the horizontal baseline in each search window, and the downward and upward trends in the SpO_2_, the other sides of the triangle; Third, S= ${\sum }_{i=1}^{n}{S}_{i}$, which is the sum of all respiratory events in the similar triangle of the specific search window, where i is the number of apnea or hypopnea events during the night; Fourth, the total area is divided by the total night recording time to yield the SASHB in units of %min/h; Fifth, a SASHB of 40% min/h corresponds to a 4% reduction in SpO_2_ below baseline for 10 min during every hour of sleep or a 5% reduction below baseline for 8 min every hour.

The calculation of SASHB relies on computer-based analysis rather than manual operation. In this study, the calculation of the SASHB index is rooted in laboratory test data, encompassing nasal airflow and blood oxygen saturation trend maps. We developed the operation program of SASHB by using MATLAB (MathWorks, R2018a, USA), and this software can realize the batch processing of the original SpO_2_ data. A description of the quality control criteria for the SpO_2_ trend graph (recording duration and artifact management) and details of the original calculation codes for SASHB were provided in the supplementary material.

### Statistical analysis

Data are presented as mean values ± standard deviation (SD) for continuous variables and percentages for categorical variables. Descriptive statistics were computed across the quartiles of SASHB. Inter-group differences in descriptive statistics were examined using analysis of variance (ANOVA) for continuous variables and chi-square tests for categorical variables. A polynomial linear trend test was used to evaluate linear trends across SASHB quartiles for continuous variables, and a linear-by-linear association test was applied for dichotomous variables. The treatment of missing data in the dataset is done using the maximum likelihood estimate method, where missing values are estimated from the marginal distribution of the observations.

Stepwise multiple regression analyses were executed separately to explore the independent associations of SASHB, AHI, CT90 with glucose metabolism indicators (FBG, FIN, HOMA-IR) and lipid profiles including TC, LDL-c, HDL-c, TG, apoA-I, apoB and apoE. Binary logistic regression analyses were employed to determine risk factors for hyperglycemia, hyperinsulinemia, abnormal HOMA-IR, hyper-total cholesterolemia, hyper-LDL cholesterolemia, hypo-HDL cholesterolemia, and hypertriglyceridemia. Linear trends were assessed by examining the median SASHB value for each quartile and conducting the overall F-test for that value. Additionally, odds ratios (ORs) and 95% confidence intervals (CIs) were also computed. Importantly, the statistical analysis was preceded using collinearity diagnostics to eliminate potential multicollinearity among variables. The two steps of the collinearity analyses were: (1) a preliminary analysis using a Spearman correlation and (2) collinearity diagnostics to determine the selected covariates in the multivariate linear regression analyses. For detail, please see Supplementary (Tables S28-S30). Following the collinearity diagnosis, Model 1 was adjusted for age, and BMI as continuous variables, as well as sex as categorical variables. Model 2 included the following covariates: age, BMI as a continuous variable; and sex, hypertension, smoking status, and alcohol consumption as categorical variables. Furthermore, Model 3 further added the MAI to Model 2. Of note, SASHB and CT90 were examined as both a classified variable according to its quartiles as well as a continuous one. These yielded similar conclusions, and we presented only the results employing the continuous variables in linear regression analyses and the quartile variables in logistic regression analyses for simplicity. All statistical analyses employed SPSS ver. 26.0 (SPSS Inc., Chicago, IL, USA). Statistical significance was defined as a bilateral *p*-value < 0.05.

## Results

### Baseline characteristics and univariate analysis

**Fig. 3:**
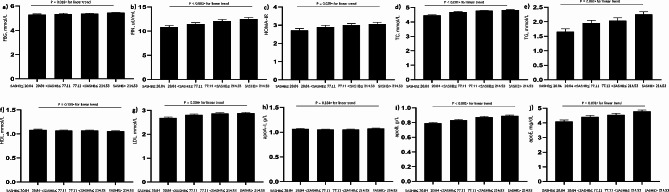
Adjusted mean values of the glucose and lipid levels in model 1. **(a)** FBG - SASHB; **(b)** FIN - SASHB; **(c)** HOMA-IR - SASHB; **(d)** TC - SASHB; **(e)** TG - SASHB; **(f)** HDL - SASHB; **(g)** LDL - SASHB; **(h)** apoA-I - SASHB; **(i)** apoB - SASHB; and **(j)** apoE - SASHB. **Abbreviations**: The data were adjusted for age, body mass index (BMI), and sex. FBG: Fasting blood glucose; FIN: Fasting insulin; HOMA-IR: Homeostasis model assessment of insulin resistance; TC: Total cholesterol; TG: Total triglycerides; HDL-C: High-density lipoprotein cholesterol; LDL-C: Low-density lipoprotein cholesterol; apoA-I: apolipoprotein A-I; apoB: apolipoprotein B; apoE: apolipoprotein E; SASHB: Sleep Apnea-Specific Hypoxic Burden

In total, 2,173 patients with suspected OSA were enrolled in this study. Of these, 1634 were male and 539 were female. Participants were categorized by SASHB quartiles (≤20.84, 20.84–77.11, 77.11–214.53, and > 214.53). Demographic characteristics (age, height, BMI, NC, WC, HC, NHR, WHR) and sleep indices (AHI, MAI) differed significantly across SASHB quartiles (all P for trend < 0.001; Table 1); Continuous variables such as FBG, FIN, HOMA-IR, TC, TG, HDL-C, LDL-C, apoB and apoE were also differed across the SASHB quartiles (all P for trend < 0.001; Table 1). Specifically, a positive dose-response relationship was observed between SASHB and FBG, FIN, HOMA-IR, TC, LDL-c, and TG levels, while conversely, a negative dose-response relationship was observed between SASHB and HDL-c levels (Fig. 3). Furthermore, the prevalence rates of Hyperglycemia, Hyperinsulinemia, insulin resistance, Hyper-total cholesterolemia, Hypo-HDL cholesterolemia, Hyper-LDL cholesterolemia, and Hyper-triglyceridemia increased with the SASHB quartile from 14.90 to 33.70%, 24.50–50.20%, 30.60–60.30%, 17.90–36.40%, 42.20–57.70%, 15.80–30.20%, and 26.10–55.00%, respectively (linear trends, *p* < 0.001) (Table 1).

**Table 1:** Characteristics, sleep parameters, and biochemical indicators of patients by SASHB quartile

Characteristic	SASHB≤20.84	20.84 < SASHB≤77.11	77.11 < SASHB≤214.53	SASHB > 214.53	*P*-value for trend^a^
No. patients	546	545	546	536	—
Demographics					
Age, y	38.04 ± 14.08	41.31 ± 13.24	45.29 ± 12.87	44.90 ± 12.35	< 0.001
Male, %	62.94	74.95	79.52	83.06	< 0.001
Height, m	1.67 ± 0.11	1.70 ± 0.09	1.70 ± 0.08	1.70 ± 0.07	< 0.001
BMI, kg/m^2^	23.88 ± 4.06	25.50 ± 3.93	26.33 ± 3.72	27.31 ± 3.21	< 0.001
NC, cm	36.08 ± 4.1	37.82 ± 3.9	38.78 ± 3.51	39.81 ± 3.11	< 0.001
WC, cm	85.85 ± 12.33	91.52 ± 10.78	94.55 ± 10.11	97.18 ± 8.56	< 0.001
HC, cm	95.96 ± 9.53	98.94 ± 8.82	100.81 ± 7.43	101.89 ± 6.63	< 0.001
NHR	0.22 ± 0.02	0.22 ± 0.02	0.23 ± 0.02	0.23 ± 0.02	< 0.001
WHR	0.89 ± 0.07	0.92 ± 0.06	0.94 ± 0.06	0.95 ± 0.06	< 0.001
MAP, mmHg	93.26 ± 10.90	93.53 ± 12.49	95.32 ± 11.16	97.34 ± 12.14	< 0.001
Sleep parameters					
AHI	4.71 ± 10.84	11.29 ± 10.38	28.35 ± 14.41	49.58 ± 17.34	< 0.001
MAI	15.70 ± 14.62	19.06 ± 16.32	24.23 ± 18.11	31.30 ± 19.37	< 0.001
Biochemical indicators					
Glucometabolism					
FBG, mmol/L	5.08 ± 1.0	5.28 ± 1.08	5.43 ± 1.22	5.61 ± 1.25	< 0.001
FIN,$uU/\text{mL}$	9.76 ± 6.95	11.4 ± 7.71	12.26 ± 7.76	13.91 ± 7.69	< 0.001
HOMA-IR	2.31 ± 2.05	2.81 ± 2.57	3.07 ± 2.49	3.55 ± 2.39	< 0.001
Hyperglycemia, %	14.90	21.40	24.70	33.70	< 0.001
Hyperinsulinemia, %	24.50	34.80	38.00	50.20	< 0.001
HOMA-IR ≥ 2.5, %	30.60	43.50	47.00	60.30	< 0.001
Lipid profiles					
TC, mmol/L	4.44 ± 0.95	4.68 ± 0.97	4.80 ± 0.90	4.86 ± 0.93	< 0.001
TG, mmol/L	1.47 ± 1.16	1.89 ± 2.05	2.04 ± 1.95	2.38 ± 2.32	< 0.001
HDL-C, mmol/L	1.14 ± 0.3	1.08 ± 0.27	1.06 ± 0.26	1.02 ± 0.23	< 0.001
LDL-C, mmol/L	2.67 ± 0.8	2.83 ± 0.82	2.91 ± 0.77	2.94 ± 0.82	< 0.001
apoA-I, g/L	1.08 ± 0.19	1.05 ± 0.17	1.05 ± 0.19	1.05 ± 0.19	0.033
apoB, g/L	0.77 ± 0.22	0.83 ± 0.20	0.88 ± 0.24	0.91 ± 0.22	< 0.001
apoE, mg/dL	4.06 ± 1.44	4.41 ± 1.89	4.53 ± 1.97	4.84 ± 2.36	< 0.001
Hyper TC, %	17.90	26.80	30.80	36.40	< 0.001
Hyper TG, %	26.10	37.10	45.20	55.00	< 0.001
Hypo HDL-C, %	42.20	50.40	50.00	57.70	< 0.001
Hyper LDL-C, %	15.80	24.80	25.40	30.20	< 0.001

**Notes**: Data are presented as mean values ± SD or percentages

^a^Tested by the polynomial linear trend test for continuous variables and the linear-by-linear association test for dichotomous variables

**Abbreviations:** SASHB, sleep apnea-specific hypoxic burden; BMI, body mass index; NC, neck circumference; WC, waist circumference; HC, hip circumference; NHR, the ratio of neck circumference to height; WHR, the ratio of waist circumference; MAP, mean artierial pressure; MAI, microarousal index; AHI, apnea hypopnea index; FBG, fasting blood glucose; FIN, fasting insulin; HOMA-IR, homeostasis model assessment of insulin resistance; TC, Total cholesterol; TG, Total triglycerides; HDL-C, High-density lipoprotein cholesterol; LDL-C, Low-density lipoprotein cholesterol; apoA-I, apolipoprotein A-I; apoB, apolipoprotein B; apoE, apolipoprotein E

### Relationship between SASHB and glucose metabolism

**Fig. 4:**
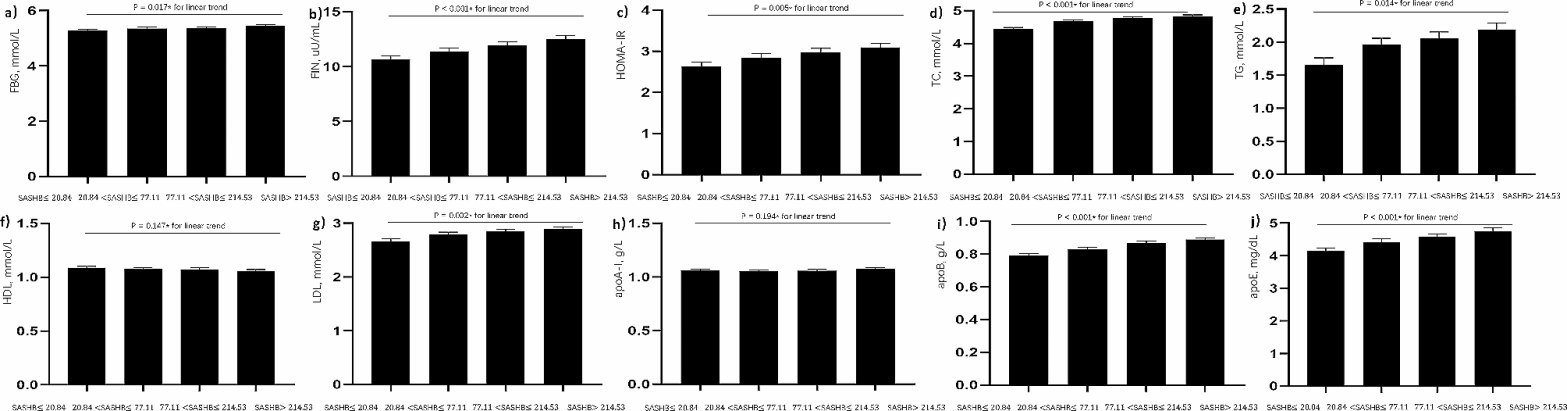
Adjusted mean values of the glucose and lipid levels in model 2. **(a)** FBG - SASHB; **(b)** FIN - SASHB; **(c)** HOMA-IR - SASHB; **(d)** TC - SASHB; **(e)** TG - SASHB; **(f)** HDL - SASHB; **(g)** LDL - SASHB; **(h)** apoA-I - SASHB; **(i)** apoB - SASHB; and **(j)** apoE - SASHB. **Abbreviations**: The data were adjusted for age, body mass index (BMI), sex, smoking status, mean artery pressure, and alcohol consumption. FBG: Fasting blood glucose; FIN: Fasting insulin; HOMA-IR: Homeostasis model assessment of insulin resistance; TC: Total cholesterol; TG: Total triglycerides; HDL-C: High-density lipoprotein cholesterol; LDL-C: Low-density lipoprotein cholesterol; apoA-I: apolipoprotein A-I; apoB: apolipoprotein B; apoE: apolipoprotein E; SASHB: Sleep Apnea-Specific Hypoxic Burden

**Fig. 5:**
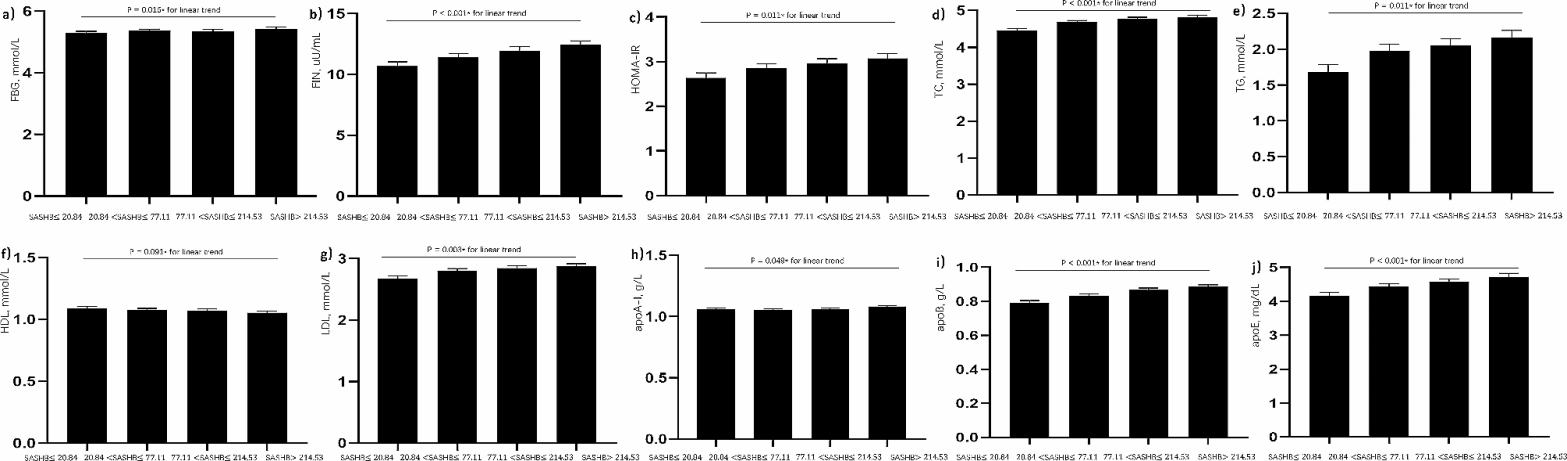
Adjusted mean values of the glucose and lipid levels in model 3. **(a)** FBG - SASHB; **(b)** FIN - SASHB; **(c)** HOMA-IR - SASHB; **(d)** TC - SASHB; **(e)** TG - SASHB; **(f)** HDL - SASHB; **(g)** LDL - SASHB; **(h)** apoA-I - SASHB; **(i)** apoB - SASHB; and **(j)** apoE - SASHB. **Abbreviations**: The data were adjusted for age, body mass index (BMI), sex, smoking status, mean artery pressure, alcohol consumption and microarousal index. FBG: Fasting blood glucose; FIN: Fasting insulin; HOMA-IR: Homeostasis model assessment of insulin resistance; TC: Total cholesterol; TG: Total triglycerides; HDL-C: High-density lipoprotein cholesterol; LDL-C: Low-density lipoprotein cholesterol; apoA-I: apolipoprotein A-I; apoB: apolipoprotein B; apoE: apolipoprotein E; SASHB: Sleep Apnea-Specific Hypoxic Burden

After adjusting for age, gender, BMI, MAP, smoking status, alcohol consumption and MAI, the SASHB was found to be independently associated with FBG (β = 0.058, *P* = 0.017), FIN (β = 0.073, *P* < 0.001), and the HOMA-IR (β = 0.058, *P* = 0.005) (Table S1, Model 3, Figs. 4 and 5).

As shown in Table S7, after adjusting for age, gender, and BMI in Model 1, as well as accounting for the MAP, smoking status, and alcohol consumption in Model 2, logistic regression models were employed to assess the association between the SASHB and abnormal glucose metabolism (i.e., hyperglycemia, hyperinsulinemia, and an abnormal HOMA-IR). Upon the incorporation of the MAI into Model 2, the ORs (95% CI) for hyperinsulinemia and abnormal HOMA-IR remained significant in the linear trend test, with ORs (95% CI) for SASHB quartiles being 1 (reference), hyperinsulinemia{1.527 (1.077, 2.166), 1.545 (1.083, 2.204), and 2.024 (1.400, 2.926), respectively (*p* < 0.001 for a linear trend)}; abnormal HOMA-IR {1.510 (1.085, 2.104), 1.492(1.064, 2.092), 1.937(1.356, 2.767), respectively (*p* = 0.001 for a linear trend)}.

### Relationship between SASHB and lipid profile

Upon adjusting for age, gender, BMI, MAP, smoking status, alcohol consumption and MAI, the SASHB was found to be independently associated with TC (β = 0.100, *P* < 0.001), TG (β = 0.063, *P* = 0.011), LDL-C (β = 0.075, *P* = 0.003), apoB (β = 0.136, *P* < 0.001) and apoE (β = 0.088, *P* < 0.001) (Table S4, Model 3, Figs. 4 and 5).

Logistic regression analyses revealed positive associations between all abnormal lipid metabolism ORs (95% CI) and increasing SASHB quartiles. These results persisted even after the MAI was incorporated into the model, with ORs (95% CI) of 1 (reference), 1.762 (1.243, 2.499), 1.998 (1.399, 2.856), and 2.708 (1.871, 3.919) (*P* < 0.001 for a linear trend) for Hyper-total cholesterolemia; ORs (95% CI) of 1 (reference), 1.663 (1.156, 2.392), 1.695 (1.164, 2.467), and 2.316 (1.574, 3.407) (*p* < 0.001 for a linear trend) for Hyper-LDL cholesterolemia; and ORs (95% CI) of 1 (reference), 1.471 (1.078, 2.007), 1.773 (1.293, 2.433), and 2.099 (1.505, 2.928) (*p* < 0.001 for a linear trend), respectively, for Hyper-triglyceridemia across SASHB quartiles (Table S7, Model 3).

### Relationship between AHI, CT90 and glycolipid metabolism

Logistic regression models were employed to assess the association between the AHI and abnormal glucose and lipid metabolism. Within Model 3, significant positive linear trends were observed for abnormal glucose and lipid metabolism (Hyperinsulinemia, HOMA-IR ≥ 2.5, Hyper-total cholesterolemia, Hyper-LDL cholesterolemia, Hyper-triglyceridemia) ORs and 95% CIs with increasing AHI quartiles (all P for trend≤0.001). Specific ORs (95% CI) are shown in the supplemental file Table S8. Similarly, we analyzed CT90, Logistic regression models were employed to assess the association between the CT90 and abnormal glucose and lipid metabolism (Table S9). The results of the stepwise multiple linear regression (AHI, CT90) of glucose metabolism index in models 1, 2 and 3 are presented in the supplemental file (Table S2, 3, 5, 6).

### Association of AHI, CT90 and SASHB with glycolipid metabolism in male and female concentration

In the male subset, Logistic regression models were employed to assess the association between the SASHB and abnormal glucose and lipid metabolism. Within Model 3, significant positive linear trends were observed for abnormal glycolipid metabolism (hyperinsulinemia, abnormal HOMA-IR, Hyper-total cholesterolemia, Hyper-LDL cholesterolemia) ORs and 95% CIs with increasing SASHB quartiles (Table S16). The results of the AHI and CT90 are shown in supplemental file Tables 17 and 18.

In the female subset, Logistic regression models were employed to assess the association between the SASHB and abnormal glucose and lipid metabolism. Within Model 3, significant positive linear trends were observed for abnormal glycolipid metabolism (hyperinsulinemia, Hyperinsulinemia, Hyper-triglyceridemia) ORs and 95% CIs with increasing SASHB quartiles (Table S25). The results of the AHI and CT90 are shown in supplemental file Tables 26 and 27.

In the men’s and female’s subset, the results of the stepwise multiple linear regression (SASHB, AHI, CT90) of glucose metabolism index in models 1, 2 and 3 are presented in the supplemental file (Table S2, 3, 5, 6).

## Discussion

The present study demonstrated independent associations between SASHB and abnormal glucose as well as lipid metabolism with substantial sample, objective PSG data, and rigorous multivariate adjustments. Our findings indicate a positive linear trend for the risk of hyperinsulinemia and abnormal HOMA-IR across SASHB quartiles in abnormal glucose metabolism after adjusting for multiple variables; In terms of abnormal lipid metabolism, we observed a positive linear trend for risk of Hyper-total cholesterolemia, Hyper-LDL cholesterolemia and Hyper-triglyceridemia across SASHB quartiles after adjusting for multiple variables.

Because SpO_2_ is readily available from laboratory and home sleep studies, it makes sense to include the depth and duration of IH among the metrics that have predictive value for abnormalities in glycolipid metabolism. SASHB is a candidate metric designed to predict the likelihood of the occurrence of abnormalities in glycolipid metabolism by capturing the frequency of respiratory events, and the depth and duration of the hypoxia associated with them. Azarbarzin et al. [29] first explored the association between SASHB and cardiovascular mortality, this study suggested that SASHB can be considered as early warning, diagnosis and prevention of cardiovascular diseases in OSA patients. Blanchard et al. examined the relationship between SASHB and incidence of new cerebrovascular events, they found that SASHB presented a higher prognostic value of cerebrovascular events when compared with other sleep variables (HR = 1.28); they also noted that SASHB may be a robust risk factor for stroke stratification in OSA [30].

Previous studies have confirmed that SASHB is one of the important early warning indicators of risk for cardiovascular morbidity and mortality, and given that abnormalities in glucose and lipid metabolism are established risk factors for cardiovascular morbidity and mortality [31], there has been an ongoing effort to find potential factors associated with glucose and lipid homeostasis. The impact of SASHB, an important early warning indicator for assessing IH in patients with OSA, on glucose metabolism and lipid metabolism remains unknown. Our findings support an independent correlation between SASHB and a range of metabolic abnormalities, including hyperinsulinemia, abnormal HOMA-IR, Hyper-cholesterolemia, Hyper -LDL cholesterolemia and Hyper-triglyceridemia. IH stimulation has been found to result in reduced insulin sensitivity and impaired glucose tolerance, with potential mechanisms of influence including activation of the sympathetic and hypothalamic-pituitary-adrenal systems, with the release of catecholamines that reduce insulin receptor sensitivity and decrease tissue insulin-mediated glucose uptake, while stimulating gluconeogenesis [32]. A previous study also found that SF could induce abnormal glucose metabolism through increased activity of the hypothalamic–pituitary–adrenal axis, resulting in higher circulating cortisol concentrations [33]. In rodent studies, SF was also associated with the development of glucose intolerance and insulin resistance [34, 35]. Sleep fragmentation is a stressor that causes the elevation of hormones such as adrenocorticotropic hormone and cortisol. These hormones play a role in lipolysis, which might affect lipid levels [36, 37] A previous study [38] reported an association between sleep fragmentation and dyslipidemia in a population of approximately 700 OSA patients. However, the included subjects in that studies were non-consecutive, which could have induced selection bias. Studies performed in animal models showed that repeated arousal from sleep could cause impaired lipid levels [39, 40].

Rodent studies demonstrated that sleep disruption and IH could lead to insulin resistance [12, 35]. Many clinical studies exploring the relationship between OSA and glucose metabolism were performed, with inconsistent results. Some studies suggested a link between OSA and abnormal glucose metabolism prior to the manifestation of diabetes [41, 42], and further demonstrated that two pathophysiological processes of OSA (SF and IH) could increase circulating glucose by decreasing insulin sensitivity and reducing glucose effectiveness [13, 43–45]. A cross-sectional study recruiting 1,834 patients with suspected OSA demonstrated that SF was independently associated with hyperinsulinemia, whereas IH was associated with hyperglycemia, hyperinsulinemia, and abnormal HOMA-IR abnormalities [46]. An epidemiologic study of 2,686 patients with suspected OSA suggests that sleep fragmentation is strongly associated with high LDL cholesterolemia in patients with OSA. It is warranted to investigate the causal relationship between sleep fragmentation and dyslipidemia, as well as the underlying mechanisms of this association further in prospective cohort studies [47].

A case-control study [48] observed inflammatory markers (IL-6, IL-8, IL-17, IL-18, MIF, Hs CRP, TNF-$\alpha$, PAI-1 and leptin) were significantly associated with OSA as compared to those without OSAs. Fang Y et al. revealed the association between autoantibodies against inflammatory factors and OSA, and the combination of auto antibodies against CRP, IL-6, IL-8 and TNF-$\alpha$may function as novel biomarker for monitoring the presence of OSA [49]. It has also been found that IH can stimulate the expression of NF-ĸB [50], leading to an increase in the expression of downstream inflammatory mediators, such as tumor necrosis factor-${\alpha }$ and interleukin-8, which results in damage to pancreatic islet cells and decreases insulin sensitivity in the liver, muscle, and adipose tissues, ultimately leading to disturbances in glucose metabolism in patients with OSA. It has also been suggested [51] that the increase in FFA levels may be mediated by hypoxia-induced up-regulation of adipose triglyceride lipase activators such as protein kinase A. OSA, in addition to affecting circulating levels of TC and FFA, also regulates lipid function through oxidative stress. oxidative stress to regulate lipid function. We speculate that the depth and duration of hypoxia may be a possible factor in the excitation of sympathetic nerve activity, expression of inflammatory mediators, and oxidative stress, although further prospective studies are needed to elucidate this potential relationship.

Combining the duration and depth of respiratory events and their associated desaturation may provide useful information for more precise identification and management of patients with OSA (precision medicine). For example, studies have shown that longer [52, 53] and deeper [54] apneas and hypoventilation elicit a greater cardiovascular response than shorter and milder apneas and hypoventilation. The autonomous relationship between SASHB and abnormalities of glucose and lipid metabolism observed in this study highlights the need for improvement with a focus on IH when developing novel treatment strategies for OSA patients with comorbid abnormalities of glucose metabolism. For example, when considering oxygen therapy for OSA, the frequency, depth and duration of oxygen were all important. This integrated approach has the potential to improve the prognosis and outcome of OSA patients with comorbid glucose metabolism abnormalities.

The highlight of this study is that all OSA indices were collected by laboratory-based PSG monitoring rather than surrogate measures (e.g., witnessed apnea or portable PSG). Additionally, we meticulously excluded individuals undergoing treatment with hypoglycemic and lipid-lowering medications. Finally, the substantial sample size and adjustment for confounding factors enhance the accuracy and credibility of our findings. Despite these merits, our study carries several limitations that merit discussion in the interpretation of our results. First, the present report is limited by the fact that it was based on clinical samples and observational research, and could not provide the causative evidence. Second, diet and physical activity are two important factors that influence glucose and lipid metabolism. Although only residents in east China with roughly analogous lifestyles were enrolled, and we strictly excluded patients who had been treated with hypoglycemic and lipid-lowering drugs, not controlling these two confounding factors is a potential limitation of our study. Third, the non-community-based prospective design of our study is a limitation worth noting. Fourth, these data are valid only for the examined population. Fifth, Morphological changes (android obesity) also play a major role in explaining both the higher hypoxic burden and the dysmetabolisms. However, we did not have the classic morphological co-variates of the metabolic syndrome been correctly entered into the model. Sixth, currently the calculation of the SASHB is based on the identification of desaturating events from ventilatory traces and qualified respiratory events. Other oximetric techniques propose to do without the detection of respiratory events and are perhaps more interesting.

## Conclusion

In conclusion, our study revealed a positive linear trend for risk of hyperinsulinemia and HOMA-IR across SASHB quartiles; Similarly, concerning abnormal lipid metabolism, we observed a positive linear trend for risk of Hyper-total cholesterolemia, Hyper-LDL cholesterolemia and Hyper-triglyceridemia across SASHB quartiles. These findings underscore the imperative need to delve deeper into the causal connection between hypoxic burden and abnormal glucose and lipid metabolism. Future research endeavors should focus on elucidating the underlying mechanisms of such an association through prospective cohort studies.

### Electronic supplementary material

Below is the link to the electronic supplementary material.

Supplementary Material 1

## Data Availability

No datasets were generated or analysed during the current study.

## Abbreviations

OSA: Obstructive sleep apnea
SASHB: Sleep apnea-specific hypoxic burden
FBG: Fasting blood glucose
FIN: Fasting insulin
TC: Total cholesterol
TG: Total triglycerides
HDL-C: High-density lipoprotein cholesterol
LDL-C: Low-density lipoprotein cholesterol
apoA-I: Apolipoprotein A-I
apoB: Apolipoprotein B
apoE: Apolipoprotein E
IH: Intermittent hypoxia
PSG: Polysomnography
EOG: Electrooculogram
EMG: Electromyogram
AASM: American Academy of Sleep Medicine
REM: Rapid eye movement
MAI: Microarousal index
AHI: Apnea hypopnea index
HOMA-IR: Homeostasis model assessment of insulin resistance
NC: Neck circumference, WC, waist circumference
HC: Hip circumference
BP: Blood pressure
BMI: Body mass index
MAP: Mean arterial pressure
MAI: Microarousal index
