# Supplementary material for: Independent relationship between sleep apnea-specific hypoxic burden and glucolipid metabolism disorder: a cross-sectional study
Source: Respir Res. 2024 May 18;25:214. doi: 10.1186/s12931-024-02846-7 (PMC11102635; doi:10.1186/s12931-024-02846-7)
Supplement: Supplementary file 1 — Supplementary Material 1 [file 12931_2024_2846_MOESM1_ESM.docx]

**Supplemental file**

**Table S1** Stepwise multiple linear regression **(SASHB)** for glucose metabolism index in model 1, 2 and 3

| Variable | Reference | FBG, mmol/L | FIN,$u\text{U}/\text{mL}$ | HOMA-IR |
| --- | --- | --- | --- | --- |
| Model 1 | | | | |
| Age, y |  | 0.189(0.002)^a^ | -0.079(0.013)^a^ | — |
| BMI |  | 0.238(0.007)^a^ | 0.438(0.044)^a^ | 0.416(0.015)^a^ |
| Sex | Male | — | — | — |
| SASHB |  | 0.055(0.002)^c^ | 0.074(0.011)^a^ | 0.050(0.004)^c^ |
| Model 2 | | | | |
| Age, y |  | 0.189(0.002)^a^ | -0.089(0.013)^a^ | — |
| BMI |  | 0.247(0.008)^a^ | 0.442(0.045)^a^ | 0.433(0.015)^a^ |
| Sex | Male | — | — | — |
| Smoking status | non-current smoker | — | — | — |
| Alcohol consumption | non-current drinker | — | 0.044(1.088)^c^ | — |
| MAP |  | — | — | — |
| SASHB |  | 0.057(0.002)^c^ | 0.081(0.012)^a^ | 0.065(0.004)^b^ |
| Model 3 |  |  |  |  |
| Age, y |  | 0.188(0.002)^a^ | -0.084(0.013)^a^ | — |
| BMI |  | 0.246(0.008)^a^ | 0.448(0.044)^a^ | 0.437(0.014)^a^ |
| Sex | Male | — | — | — |
| Smoking status | non-current smoker | — | — | — |
| Alcohol consumption | non-current drinker | — | 0.046(1.057)^c^ | — |
| MAP |  | — | — | — |
| MAI |  | — | — | — |
| SASHB |  | 0.058(0.002)^c^ | 0.073(0.011)^a^ | 0.058(0.004)^c^ |

**Notes:** Data are presented as β (SE[β]). Model 1 adjusted for age, body mass index (BMI), sex and plus Smoking status, Alcohol consumption, mean artierial pressure in model 2 and plus MAI in model 3. ^a^p<0.001, ^b^p<0.01, ^c^p<0.05.

**Abbreviations:** BMI, body mass index; SASHB, sleep apnea-specific hypoxic burden; MAP, mean artierial pressure; MAI, microarousal index; FBG, fasting blood glucose; FIN, fasting insulin; HOMA-IR, homeostasis model assessment of insulin resistance.

**Table S2** Stepwise multiple linear regression **(AHI)** for glucose metabolism index in model 1, 2 and 3

| Variable | Reference | FBG, mmol/L | FIN,$u\text{U}/\text{mL}$ | HOMA-IR |
| --- | --- | --- | --- | --- |
| Model 1 | | | | |
| Age, y |  | 0.182(0.002)^a^ | -0.082(0.013)^a^ | — |
| BMI |  | 0.223(0.007)^a^ | 0.429(0.045)^a^ | 0.405(0.015)^a^ |
| Sex | Male | — | — | — |
| AHI |  | 0.090(0.001)^a^ | 0.089(0.008)^a^ | 0.073(0.003)^a^ |
| Model 2 | | | | |
| Age, y |  | 0.183(0.002)^a^ | -0.093(0.013)^a^ | — |
| BMI |  | 0.233(0.008)^a^ | 0.434(0.046)^a^ | 0.422(0.015)^a^ |
| Sex | Male | — | — | — |
| Smoking status | non-current smoker | — | — | — |
| Alcohol consumption | non-current drinker | — | — | — |
| MAP |  | — | — | — |
| AHI |  | 0.088(0.001)^a^ | 0.105(0.008)^a^ | 0.086(0.003)^a^ |
| Model 3 |  |  |  |  |
| Age, y |  | 0.182(0.002)^a^ | -0.090(0.013)^a^ | — |
| BMI |  | 0.232(0.008)^a^ | 0.435(0.045)^a^ | 0.427(0.014)^a^ |
| Sex | Male | — | — | 0.048(0.118)^c^ |
| Smoking status | non-current smoker | — | — | — |
| Alcohol consumption | non-current drinker | — | — | — |
| MAP |  | — | — | — |
| MAI |  | — | — | — |
| AHI |  | 0.089(0.001)^a^ | 0.110(0.008)^a^ | 0.100(0.002)^a^ |

**Notes:** Data are presented as β (SE[β]). Model 1 adjusted for age, body mass index (BMI), sex and plus Smoking status, Alcohol consumption, mean artierial pressure in model 2 and plus MAI in model 3. ^a^p<0.001, ^b^p<0.01, ^c^p<0.05.

**Abbreviations:** BMI, body mass index; AHI, apnea-hyponea index; MAP, mean artierial pressure; MAI, microarousal index; FBG, fasting blood glucose; FIN, fasting insulin; HOMA-IR, homeostasis model assessment of insulin resistance.

**Table S3** Stepwise multiple linear regression **(CT90)** for glucose metabolism index in model 1, 2 and 3

| Variable | Reference | FBG, mmol/L | FIN,$u\text{U}/\text{mL}$ | HOMA-IR |
| --- | --- | --- | --- | --- |
| Model 1 | | | | |
| Age, y |  | 0.193(0.002)^a^ | -0.070(0.013)^a^ | — |
| BMI |  | 0.235(0.007)^a^ | 0.458(0.042)^a^ | 0.429(0.014)^a^ |
| Sex | Male | — | — | — |
| CT90 |  | 0.065(0.004)^b^ | — | — |
| Model 2 | | | | |
| Age, y |  | 0.193(0.002)^a^ | -0.081(0.013)^a^ | — |
| BMI |  | 0.247(0.008)^a^ | 0.451(0.045)^a^ | 0.451(0.014)^a^ |
| Sex | Male | — | — | — |
| Smoking status | non-current smoker | — | — | — |
| Alcohol consumption | non-current drinker | — | 0.050(1.087)^c^ | — |
| MAP |  | — | — | — |
| CT90 |  | 0.060(0.004)^c^ | 0.048(0.025)^c^ | — |
| Model 3 |  |  |  |  |
| Age, y |  | 0.193(0.002)^a^ | -0.077(0.013)^a^ | — |
| BMI |  | 0.245(0.008)^a^ | 0.454(0.044)^a^ | 0.454(0.014)^a^ |
| Sex | Male | — | — | — |
| Smoking status | non-current smoker | — | — | — |
| Alcohol consumption | non-current drinker | — | 0.051(1.055)^c^ | — |
| MAP |  | — | — | — |
| MAI |  | — | — | — |
| CT90 |  | 0.063(0.004)^b^ | 0.049(0.024)^c^ | — |

**Notes:** Data are presented as β (SE[β]). Model 1 adjusted for age, body mass index (BMI), sex and plus Smoking status, Alcohol consumption, mean artierial pressure in model 2 and plus MAI in model 3. ^a^p<0.001, ^b^p<0.01, ^c^p<0.05.

**Abbreviations:** BMI, body mass index; CT90, percentage of total sleep time with SpO_2_<90%; MAP, mean artierial pressure; MAI, microarousal index; FBG, fasting blood glucose; FIN, fasting insulin; HOMA-IR, homeostasis model assessment of insulin resistance.

**Table S4** Stepwise multiple linear regression **(SASHB)** for Lipid profiles in model 1, 2 and 3

| Variable | Reference | TC, mmol/L | TG, mmol/L | HDL-C, mmol/L | LDL-C, mmol/L | apoA-I, g/L | apoB, g/L | apoE, mg/dL |
| --- | --- | --- | --- | --- | --- | --- | --- | --- |
| Model 1 | | | | | | | | |
| Age, y |  | 0.095(0.002)^a^ | — | 0.050(0.001)^c^ | 0.053(0.002)^c^ | 0.130(0.001)^a^ | 0.088(0.001)^a^ | — |
| BMI |  | 0.093(0.006)^a^ | 0.160(0.013)^a^ | -0.233(0.002)^a^ | 0.087(0.005)^a^ | -0.115(0.001)^a^ | 0.184(0.001)^a^ | 0.157(0.013)^a^ |
| Sex | Male | — | -0.066(0.109)^b^ | 0.275(0.014)^a^ | — | 0.220(0.01)^a^ | — | 0.084(0.111)^a^ |
| SASHB |  | 0.097(0.002)^a^ | 0.074(0.003)^b^ | — | 0.070(0.001)^b^ | — | 0.141(0.001)^a^ | 0.093(0.003)^a^ |
| Model 2 | | | | | | | | |
| Age,y |  | 0.100(0.002)^a^ | — | 0.053(0.001)^c^ | 0.059(0.002)^c^ | 0.139(0.001)^a^ | 0.088(0.001)^a^ | — |
| BMI |  | 0.094(0.006)^a^ | 0.159(0.013)^a^ | -0.229(0.002)^a^ | 0.092(0.005)^a^ | -0.117(0.001)^a^ | 0.184(0.002)^a^ | 0.159(0.014)^a^ |
| Sex | Male | — | -0.077(0.112)^a^ | 0.283(0.014)^a^ | — | 0.221(0.011)^a^ | — | 0.074(0.114)^b^ |
| Smoking status | non-current smoker | — | — | 0.048(0.124)^c^ | — | — | — | — |
| Alcohol consumption | non-current drinker | — | 0.049(0.317)^c^ | — | — | — | 0.062(0.036)^b^ | — |
| MAP |  | — | — | — | — | — | — | — |
| SASHB |  | 0.100(0.002)^a^ | 0.062(0.003)^c^ | — | 0.077(0.001)^b^ | — | 0.137(0.001)^a^ | 0.087(0.003)^a^ |
| Model 3 | | | | | | | | |
| Age,y |  | 0.100(0.002)^a^ | — | 0.053(0.001)^c^ | 0.060(0.002)^c^ | 0.137(0.001)^a^ | 0.089(0.001)^a^ | — |
| BMI |  | 0.095(0.006)^a^ | 0.160(0.013)^a^ | -0.228(0.002)^a^ | 0.092(0.005)^a^ | -0.120(0.001)^a^ | 0.184(0.002)^a^ | 0.160(0.014)^a^ |
| Sex | Male | — | -0.077(0.112)^a^ | 0.282(0.014)^a^ | — | 0.221(0.011)^a^ | — | 0.073(0.114)^b^ |
| Smoking status | non-current smoker | — | — | 0.048(0.124)^c^ | — | — | — | — |
| Alcohol consumption | non-current drinker | — | 0.049(0.317)^c^ | — | — | — | 0.063(0.036)^b^ | — |
| MAP |  | — | — | — | — | — | — | — |
| MAI |  | — | — | — | — | -0.069(0.001)^b^ | — | — |
| SASHB |  | 0.100(0.002)^a^ | 0.063(0.003)^c^ | — | 0.075(0.001)^b^ | 0.051(0.001)^c^ | 0.136(0.001)^a^ | 0.088(0.003)^a^ |

**Notes:** Data are presented as β (SE[β]). Model 1 adjusted for age, body mass index (BMI), sex and plus Smoking status, Alcohol consumption, mean artierial pressure in model 2 and plus MAI in model 3. ^a^p<0.001, ^b^p<0.01, ^c^p<0.05.

**Abbreviations:** BMI, body mass index; SASHB, sleep apnea-specific hypoxic burden; MAP, mean artierial pressure; MAI, microarousal index;

TC, Total cholesterol; TG, Total triglycerides; HDL-C, High-density lipoprotein cholesterol; LDL-C, Low-density lipoprotein cholesterol; apoA-I, apolipoprotein A-I; apoB, apolipoprotein B; apoE, apolipoprotein

**Table S5** Stepwise multiple linear regression **(AHI)** for Lipid profiles in model 1, 2 and 3

| Variable | Reference | TC, mmol/L | TG, mmol/L | HDL-C, mmol/L | LDL-C, mmol/L | apoA-I, g/L | apoB, g/L | apoE, mg/dL |
| --- | --- | --- | --- | --- | --- | --- | --- | --- |
| Model 1 | | | | | | | | |
| Age, y |  | 0.088(0.002)^a^ | — | 0.059(0.002)^b^ | 0.048(0.002)^c^ | 0.130(0.002)^a^ | 0.080(0.001)^a^ | — |
| BMI |  | 0.075(0.006)^b^ | 0.143(0.013)^a^ | -0.217(0.002)^a^ | 0.075(0.005)^b^ | -0.115(0.001)^a^ | 0.163(0.001)^a^ | 0.137(0.013)^a^ |
| Sex | Male | — | -0.058(0.109)^c^ | 0.267(0.014)^a^ | — | 0.220(0.010)^a^ | — | 0.092(0.110)^a^ |
| AHI |  | 0.133(0.001)^a^ | 0.119(0.002)^a^ | -0.052(0.002)^c^ | 0.096(0.001)^a^ | — | 0.178(0.001)^a^ | 0.141(0.002)^a^ |
| Model 2 | | | | | | | | |
| Age,y |  | 0.093(0.002)^a^ | — | 0.064(0.002)^b^ | 0.054(0.002)^c^ | 0.139(0.001)^a^ | 0.081(0.001)^a^ | — |
| BMI |  | 0.077(0.006)^b^ | 0.144(0.013)^a^ | -0.211(0.002)^a^ | 0.079(0.005)^b^ | -0.117(0.001)^a^ | 0.164(0.002)^a^ | 0.140(0.014)^a^ |
| Sex | Male | — | -0.073(0.111)^b^ | 0.272(0.015)^a^ | — | 0.221(0.011)^a^ | — | 0.082(0.114)^a^ |
| Smoking status | non-current smoker | — | — | 0.045(0.124)^c^ | — | — | — | — |
| Alcohol consumption | non-current drinker | — | — | — | — | — | 0.057(0.036)^c^ | — |
| MAP |  | — | — | — | — | — | — | — |
| AHI |  | 0.135(0.001)^a^ | 0.110(0.002)^a^ | -0.056(0.002)^c^ | 0.103(0.001)^a^ | — | 0.173(0.001)^a^ | 0.142(0.002)^a^ |
| Model 3 | | | | | | | | |
| Age,y |  | 0.093(0.002)^a^ | — | 0.063(0.001)^b^ | 0.054(0.002)^c^ | 0.134(0.001)^a^ | 0.082(0.001)^a^ | — |
| BMI |  | 0.077(0.006)^a^ | 0.145(0.013)^a^ | -0.211(0.002)^a^ | 0.079(0.005)^b^ | -0.127(0.001)^a^ | 0.163(0.002)^a^ | 0.140(0.014)^a^ |
| Sex | Male | — | -0.073(0.112)^b^ | 0.272(0.015)^a^ | — | 0.224(0.011)^a^ | — | 0.082(0.114)^a^ |
| Smoking status | non-current smoker | — | — | 0.045(0.124)^c^ | — | — | — | — |
| Alcohol consumption | non-current drinker | — | — | — | — | — | 0.057(0.036)^c^ | — |
| MAP |  | — | — | — | — | — | — | — |
| MAI |  | — | — | — | — | -0.077(0.001)^b^ | — | — |
| AHI |  | 0.134(0.001)^a^ | 0.110(0.002)^a^ | -0.055(0.002)^c^ | 0.102(0.001)^a^ | 0.065(0.002)^c^ | 0.173(0.001)^a^ | 0.133(0.002)^a^ |

**Notes:** Data are presented as β (SE[β]). Model 1 adjusted for age, body mass index (BMI), sex and plus Smoking status, Alcohol consumption, mean artierial pressure in model 2 and plus MAI in model 3. ^a^p<0.001, ^b^p<0.01, ^c^p<0.05.

**Abbreviations:** BMI, body mass index; AHI, apnea-hyponea index; MAP, mean artierial pressure; MAI, microarousal index;

TC, Total cholesterol; TG, Total triglycerides; HDL-C, High-density lipoprotein cholesterol; LDL-C, Low-density lipoprotein cholesterol; apoA-I, apolipoprotein A-I; apoB, apolipoprotein B; apoE, apolipoprotein

**Table S6** Stepwise multiple linear regression **(CT90)** for glucose metabolism index in model 1, 2 and 3

| Variable | Reference | TC, mmol/L | TG, mmol/L | HDL-C, mmol/L | LDL-C, mmol/L | apoA-I, g/L | apoB, g/L | apoE, mg/dL |
| --- | --- | --- | --- | --- | --- | --- | --- | --- |
| Model 1 | | | | | | | | |
| Age, y |  | 0.105(0.002)^a^ | — | 0.050(0.001)^c^ | 0.059(0.002)^c^ | 0.127(0.001)^a^ | 0.110(0.001)^a^ | — |
| BMI |  | 0.103(0.006)^a^ | 0.178(0.012)^a^ | -0.233(0.002)^a^ | 0.088(0.005)^a^ | -0.128(0.001)^a^ | 0.189(0.001)^a^ | 0.162(0.013)^a^ |
| Sex | Male | — | -0.074(0.108)^a^ | 0.275(0.014)^a^ | — | 0.225(0.010)^a^ | -0.051(0.012)^c^ | 0.080(0.111)^a^ |
| CT90 |  | 0.056(0.003)^c^ | — | — | 0.064(0.003)^b^ | 0.051(0.001)^c^ | 0.093(0.001)^a^ | 0.071(0.007)^b^ |
| Model 2 | | | | | | | | |
| Age,y |  | 0.111(0.002)^a^ | — | 0.053(0.001)^c^ | 0.066(0.002)^b^ | 0.136(0.001)^a^ | 0.110(0.001)^a^ |  |
| BMI |  | 0.105(0.006)^a^ | 0.175(0.013)^a^ | -0.229(0.002)^a^ | 0.094(0.005)^a^ | -0.130(0.001)^a^ | 0.189(0.002)^a^ | 0.161(0.014)^a^ |
| Sex | Male | — | -0.085(0.111)^a^ | 0.283(0.014)^a^ | — | 0.225(0.011)^a^ | -0.048(0.013)^c^ | 0.074(0.114)^b^ |
| Smoking status | non-current smoker | — | — | 0.048(0.124)^c^ | — | — | — | — |
| Alcohol consumption | non-current drinker | — | 0.054(0.317)^c^ | — | — | — | 0.067(0.036)^b^ | 0.052(0.321)^c^ |
| MAP |  | — | — | — | — | — | — | — |
| CT90 |  | 0.060(0.003)^c^ | — | — | 0.073(0.003)^b^ | 0.049(0.001)^c^ | 0.095(0.001)^a^ | 0.067(0.007)^b^ |
| Model 3 | | | | | | | | |
| Age,y |  | 0.109(0.002)^a^ | — | 0.053(0.001)^c^ | 0.066(0.002)^b^ | 0.141(0.001)^a^ | 0.111(0.001)^a^ | — |
| BMI |  | 0.114(0.006)^a^ | 0.169(0.013)^a^ | -0.228(0.002)^a^ | 0.094(0.005)^a^ | -0.126(0.001)^a^ | 0.188(0.002)^a^ | 0.161(0.014)^a^ |
| Sex | Male | — | -0.081(0.111)^a^ | 0.282(0.014)^a^ | — | 0.220(0.011)^a^ | -0.049(0.013)^c^ | 0.073(0.114)^b^ |
| Smoking status | non-current smoker | — | — | 0.048(0.124)^c^ | — | — | — | — |
| Alcohol consumption | non-current drinker | — | 0.051(0.317)^c^ | — | — | — | 0.067(0.036)^b^ | 0.052(0.321)^c^ |
| MAP |  | — | — | — | — | — | — | — |
| MAI |  | 0.062(0.001)^c^ | 0.053(0.003)^c^ | — | — | -0.078(0.001)^b^ | — | — |
| CT90 |  | — | — | — | 0.071(0.003)^b^ | 0.076(0.001)^b^ | 0.094(0.001)^a^ | 0.068(0.007)^b^ |

**Notes:** Data are presented as β (SE[β]). Model 1 adjusted for age, body mass index (BMI), sex and plus Smoking status, Alcohol consumption, mean artierial pressure in model 2 and plus MAI in model 3. ^a^p<0.001, ^b^p<0.01, ^c^p<0.05.

**Abbreviations:** BMI, body mass index; CT90, percentage of total sleep time with SpO2<90%; MAP, mean artierial pressure; MAI, microarousal index;

TC, Total cholesterol; TG, Total triglycerides; HDL-C, High-density lipoprotein cholesterol; LDL-C, Low-density lipoprotein cholesterol; apoA-I, apolipoprotein A-I; apoB, apolipoprotein B; apoE, apolipoprotein

**Table S7** Adjusted odds ratios for abnormal glucose and lipid metabolism according to SASHB categories in models 1, 2 and 3

|  | Hyperglycemia | Hyperinsulinemia | HOMA-IR≥2.5 | Hyper-total cholesterolemia | Hyper-LDL cholesterolemia | Hypo-HDL cholesterolemia | Hyper- triglyceridemia |
| --- | --- | --- | --- | --- | --- | --- | --- |
| Adjusted OR(95% CI) in model 1 | | | | | | | |
| SASHB≤20.84 | 1 | 1 | 1 | 1 | 1 | 1 | 1 |
| 20.84 < SASHB≤77.11 | 1.235(0.866,1.761) | 1.382(0.995,1.920) | 1.363(0.996,1.864) | 1.670(1.198,2.327) | 1.648(1.160,2.340) | 1.059(0.797,1.406) | 1.395(1.036,1.879) |
| 77.11< SASHB≤214.53 | 1.054(0.741,1.501) | 1.401(1.008,1.948) | 1.378(1.006,1.887) | 1.916(1.376,2.666) | 1.729(1.216,2.458) | 0.931(0.698,1.241) | 1.764(1.312,2.372) |
| SASHB>214.53 | 1.452(1.024,2.060) | 1.806(1.295,2.520) | 1.790(1.295,2.474) | 2.414(1.728,3.373) | 2.118(1.487,3.017) | 0.988(0.733,1.332) | 2.268(1.675,3.071) |
| P-value for linear trend | 0.076 | 0.001^*^ | 0.001^*^ | <0.001^*^ | <0.001^*^ | 0.724 | <0.001^*^ |
| Adjusted OR(95% CI) in model 2 | | | | | | | |
| SASHB≤20.84 | 1 | 1 | 1 | 1 | 1 | 1 | 1 |
| 20.84 < SASHB≤77.11 | 1.199(0.824,1.743) | 1.528(1.078,2.165) | 1.488(1.070,2.070) | 1.688(1.187,2.400) | 1.641(1.131,2.379) | 1.025(0.763,1.378) | 1.437(1.052,1.964) |
| 77.11< SASHB≤214.53 | 1.025(0.704,1.495) | 1.543(1.085,2.195) | 1.468(1.052,2.051) | 1.952(1.369,2.782) | 1.707(1.171,2.488) | 0.907(0.670,1.227) | 1.762(1.287,2.412) |
| SASHB>214.53 | 1.432(0.985,2.082) | 2.031(1.422,2.901) | 1.914(1.357,2.700) | 2.648(1.850,3.791) | 2.323(1.591,3.390) | 0.953(0.695,1.306) | 2.120(1.535,2.926) |
| P-value for linear trend | 0.104 | <0.001^*^ | 0.001^*^ | <0.001^*^ | <0.001^*^ | 0.596 | <0.001^*^ |
| Adjusted OR(95% CI) in model 3 | | | | | | | |
| SASHB≤20.84 | 1 | 1 | 1 | 1 | 1 | 1 | 1 |
| 20.84 < SASHB≤77.11 | 1.216(0.835,1.771) | 1.527(1.077,2.166) | 1.510(1.085,2.104) | 1.762(1.243,2.499) | 1.663(1.156,2.392) | 1.030(0.769,1.381) | 1.471(1.078,2.007) |
| 77.11< SASHB≤214.53 | 1.036(0.708,1.516) | 1.545(1.083,2.204) | 1.492(1.064,2.092) | 1.998(1.399,2.856) | 1.695(1.164,2.467) | 0.924(0.682,1.253) | 1.773(1.293,2.433) |
| SASHB>214.53 | 1.430(0.972,2.105) | 2.024(1.400,2.926) | 1.937(1.356,2.767) | 2.708(1.871,3.919) | 2.316(1.574,3.407) | 0.980(0.708,1.356) | 2.099(1.505,2.928) |
| P-value for linear trend | 0.128 | <0.001^*^ | 0.001^*^ | <0.001^*^ | <0.001^*^ | 0.741 | <0.001^*^ |

**Notes:** ORs were adjusted for age, body mass index (BMI), sex and plus Smoking status, Alcohol consumption, mean artierial pressure in model 2 and plus MAI in model 3; P-values for linear trends were determined by examining the median SASHB value for each quartile and assessing the overall F test for the median MAI variable. *P<0.05.

**Abbreviations:** SASHB, sleep apnea-specific hypoxic burden.

**Table S8** Adjusted odds ratios for abnormal glucose and lipid metabolism according to AHI categories in models 1, 2 and 3

|  | Hyperglycemia | Hyperinsulinemia | HOMA-IR≥2.5 | Hyper-total cholesterolemia | Hyper-LDL cholesterolemia | Hypo-HDL cholesterolemia | Hyper- triglyceridemia |
| --- | --- | --- | --- | --- | --- | --- | --- |
| Adjusted OR(95% CI) in model 1 | | | | | | | |
| AHI<5 | 1 | 1 | 1 | 1 | 1 | 1 | 1 |
| 5≤AHI<15 | 1.062(0.732,1.541) | 1.477(1.051,2.076) | 1.236(0.893,1.713) | 2.012(1.414,2.863) | 2.17(1.496,3.146) | 0.885(0.657,1.192) | 1.48(1.081,2.027) |
| 15≤AHI<30 | 1.004(0.698,1.445) | 1.395(0.993,1.959) | 1.241(0.895,1.721) | 2.646(1.871,3.742) | 2.477(1.713,3.581) | 0.741(0.547,1.003) | 2.204(1.619,3.001) |
| AHI≥30 | 1.397(1.018,1.917) | 1.701(1.254,2.307) | 1.693(1.262,2.271) | 2.846(2.068,3.918) | 2.497(1.774,3.514) | 0.988(0.752,1.297) | 2.637(1.995,3.486) |
| P-value for linear trend | 0.005^*^ | 0.003^*^ | <0.001^*^ | <0.001^*^ | <0.001^*^ | 0.820 | <0.001^*^ |
| Adjusted OR(95% CI) in model 2 | | | | | | | |
| AHI<5 | 1 | 1 | 1 | 1 | 1 | 1 | 1 |
| 5≤AHI<15 | 0.984(0.663,1.46) | 1.596(1.112,2.29) | 1.292(0.916,1.822) | 2.102(1.445,3.058) | 2.405(1.617,3.575) | 0.783(0.573,1.07) | 1.391(1,1.935) |
| 15≤AHI<30 | 1.001(0.679,1.475) | 1.476(1.026,2.124) | 1.281(0.904,1.813) | 2.761(1.903,4.004) | 2.613(1.755,3.889) | 0.706(0.513,0.973) | 2.157(1.556,2.99) |
| AHI≥30 | 1.368(0.974,1.921) | 1.946(1.403,2.7) | 1.827(1.335,2.5) | 3.096(2.193,4.37) | 2.836(1.959,4.106) | 0.929(0.695,1.24) | 2.445(1.817,3.291) |
| P-value for linear trend | 0.012^*^ | 0.001^*^ | <0.001^*^ | <0.001^*^ | <0.001^*^ | 0.828 | <0.001^*^ |
| Adjusted OR(95% CI) in model 3 | | | | | | | |
| AHI<5 | 1 | 1 | 1 | 1 | 1 | 1 | 1 |
| 5≤AHI<15 | 0.984(0.663,1.459) | 1.598(1.113,2.295) | 1.295(0.918,1.828) | 2.102(1.445,3.06) | 2.413(1.622,3.589) | 0.789(0.577,1.079) | 1.393(1.001,1.939) |
| 15≤AHI<30 | 0.984(0.665,1.455) | 1.481(1.026,2.136) | 1.283(0.904,1.823) | 2.773(1.907,4.032) | 2.643(1.77,3.946) | 0.717(0.519,0.989) | 2.179(1.568,3.028) |
| AHI≥30 | 1.354(0.953,1.925) | 1.961(1.396,2.756) | 1.825(1.316,2.531) | 3.099(2.17,4.426) | 2.875(1.961,4.217) | 0.957(0.707,1.294) | 2.435(1.788,3.316) |
| P-value for linear trend | 0.018^*^ | 0.001^*^ | <0.001^*^ | <0.001^*^ | <0.001^*^ | 0.632 | <0.001^*^ |

**Notes:** ORs were adjusted for age, body mass index (BMI), sex and plus Smoking status, Alcohol consumption, mean artierial pressure in model 2 and plus MAI in model 3; P-values for linear trends were determined by examining the median AHI value for each quartile and assessing the overall F test for the median MAI variable. *P<0.05.

**Abbreviations:** AHI, apnea-hyponea index.

**Table S9** Adjusted odds ratios for abnormal glucose and lipid metabolism according to CT90 categories in models 1, 2 and 3

|  | Hyperglycemia | Hyperinsulinemia | HOMA-IR≥2.5 | Hyper-total cholesterolemia | Hyper-LDL cholesterolemia | Hypo-HDL cholesterolemia | Hyper- triglyceridemia |
| --- | --- | --- | --- | --- | --- | --- | --- |
| Adjusted OR(95% CI) in model 1 | | | | | | | |
| CT90≤0.021 | 1 | 1 | 1 | 1 | 1 | 1 | 1 |
| 0.021<CT90≤1.053 | 1.350(0.966,1.886) | 1.241(0.911,1.689) | 1.381(1.027,1.855) | 1.920(1.397,2.639) | 1.807(1.282,2.547) | 0.977(0.746,1.278) | 1.315(0.993,1.740) |
| 1.053<CT90≤3.835 | 1.075(0.737,1.569) | 1.185(0.835,1.681) | 1.137(0.811,1.596) | 2.373(1.672,3.368) | 2.651(1.832,3.836) | 0.959(0.703,1.308) | 2.092(1.529,2.861) |
| CT90>3.835 | 1.491(1.069,2.080) | 1.577(1.151,2.161) | 1.667(1.225,2.269) | 2.483(1.795,3.435) | 2.329(1.645,3.298) | 0.930(0.700,1.236) | 1.777(1.335,2.367) |
| P-value for linear trend | 0.002^*^ | 0.140 | 0.119 | 0.078 | 0.025^*^ | 0.208 | 0.042^*^ |
| Adjusted OR(95% CI) in model 2 | | | | | | | |
| CT90≤0.021 | 1 | 1 | 1 | 1 | 1 | 1 | 1 |
| 0.021<CT90≤1.053 | 1.328(0.934,1.887) | 1.358(0.980,1.882) | 1.504(1.102,2.052) | 2.002(1.429,2.804) | 1.995(1.385,2.873) | 0.935(0.707,1.238) | 1.346(1.004,1.804) |
| 1.053<CT90≤3.835 | 1.080(0.727,1.604) | 1.272(0.878,1.842) | 1.210(0.847,1.730) | 2.410(1.661,3.497) | 2.689(1.809,3.996) | 0.946(0.684,1.308) | 1.925(1.385,2.675) |
| CT90>3.835 | 1.470(1.032,2.094) | 1.794(1.283,2.507) | 1.846(1.331,2.560) | 2.778(1.964,3.929) | 2.696(1.856,3.915) | 0.895(0.664,1.208) | 1.733(1.279,2.347) |
| P-value for linear trend | 0.005^*^ | 0.057 | 0.070 | 0.032^*^ | 0.010^*^ | 0.337 | 0.090 |
| Adjusted OR(95% CI) in model 3 | | | | | | | |
| CT90≤0.021 | 1 | 1 | 1 | 1 | 1 | 1 | 1 |
| 0.021<CT90≤1.053 | 1.323(0.930,1.882) | 1.360(0.980,1.887) | 1.505(1.102,2.055) | 1.995(1.423,2.796) | 2.001(1.388,2.885) | 0.943(0.712,1.249) | 1.334(0.995,1.790) |
| 1.053<CT90≤3.835 | 1.041(0.697,1.553) | 1.273(0.877,1.849) | 1.191(0.831,1.707) | 2.418(1.661,3.518) | 2.729(1.830,4.068) | 0.948(0.683,1.315) | 1.898(1.362,2.647) |
| CT90>3.835 | 1.456(1.011,2.098) | 1.793(1.267,2.539) | 1.839(1.310,2.581) | 2.736(1.914,3.911) | 2.715(1.848,3.989) | 0.915(0.671,1.248) | 1.668(1.218,2.284) |
| P-value for linear trend | 0.005^*^ | 0.103 | 0.123 | 0.091 | 0.020^*^ | 0.389 | 0.266 |

**Notes:** ORs were adjusted for age, body mass index (BMI), sex and plus Smoking status, Alcohol consumption, mean artierial pressure in model 2 and plus MAI in model 3; P-values for linear trends were determined by examining the median CT90 value for each quartile and assessing the overall F test for the median MAI variable. *P<0.05.

**Abbreviations:** CT90, percentage of total sleep time with SpO_2_<90%.

**Table S10** Stepwise multiple linear regression **(SASHB)** for glucose metabolism index in model 1, 2 and 3 **in Male subjects**

| Variable | Reference | FBG, mmol/L | FIN,$u\text{U}/\text{mL}$ | HOMA-IR |
| --- | --- | --- | --- | --- |
| Model 1 | | | | |
| Age, y |  | 0.204(0.002)^a^ | 0.416(0.058)^a^ | 0.405(0.018)^a^ |
| BMI |  | 0.210(0.009)^a^ | -0.086(0.016)^a^ | — |
| SASHB |  | — | 0.055(0.001)^c^ | — |
| Model 2 | | | | |
| Age, y |  | 0.201(0.002)^a^ | -0.105(0.017)^a^ | — |
| BMI |  | 0.210(0.009)^a^ | 0.412(0.060)^a^ | 0.406(0.018)^a^ |
| Smoking status | non-current smoker | — | — | — |
| Alcohol consumption | non-current drinker | — | — | — |
| MAP |  | — | — | — |
| SASHB |  | — | 0.072(0.001)^b^ | 0.055(0.002)^c^ |
| Model 3 |  |  |  |  |
| Age, y |  | 0.196(0.002)^a^ | -0.101(0.016)^a^ | — |
| BMI |  | 0.200(0.009)^a^ | 0.416(0.058)^a^ | 0.423(0.017)^a^ |
| Smoking status | non-current smoker | — | — | — |
| Alcohol consumption | non-current drinker | — | — | — |
| MAP |  | — | — | — |
| MAI |  | 0.071(0.002)^b^ | — | — |
| SASHB |  | — | 0.063(0.001)^b^ | — |

**Notes:** Data are presented as β (SE[β]). Model 1 adjusted for age, body mass index (BMI), sex and plus Smoking status, Alcohol consumption, mean artierial pressure in model 2 and plus MAI in model 3. ^a^p<0.001, ^b^p<0.01, ^c^p<0.05.

**Abbreviations:** BMI, body mass index; SASHB, sleep apnea-specific hypoxic burden; MAP, mean artierial pressure; MAI, microarousal index; FBG, fasting blood glucose; FIN, fasting insulin; HOMA-IR, homeostasis model assessment of insulin resistance.

**Table S11** Stepwise multiple linear regression **(AHI)** for glucose metabolism index in model 1, 2 and 3 **in Male subjects**

| Variable | Reference | FBG, mmol/L | FIN,$u\text{U}/\text{mL}$ | HOMA-IR |
| --- | --- | --- | --- | --- |
| Model 1 | | | | |
| Age, y |  | 0.195(0.002)^a^ | -0.089(0.016)^a^ | — |
| BMI |  | 0.188(0.009)^a^ | 0.408(0.059)^a^ | 0.389(0.019)^a^ |
| AHI |  | 0.073(0.001)^b^ | 0.072(0.009)^b^ | 0.053(0.003)^c^ |
| Model 2 | | | | |
| Age, y |  | 0.191(0.002)^a^ | -0.109(0.017)^a^ |  |
| BMI |  | 0.187(0.009)^a^ | 0.401(0.060)^a^ | 0.398(0.018)^a^ |
| Smoking status | non-current smoker | — | — | — |
| Alcohol consumption | non-current drinker | — | — | — |
| MAP |  | — | — | — |
| AHI |  | 0.071(0.001)^c^ | 0.093(0.009)^b^ | 0.072(0.003)^b^ |
| Model 3 |  |  |  |  |
| Age, y |  | 0.196(0.002)^a^ | -0.106(0.016)^a^ | — |
| BMI |  | 0.200(0.009)^a^ | 0.400(0.058)^a^ | 0.397(0.018)^a^ |
| Smoking status | non-current smoker | — | — | — |
| Alcohol consumption | non-current drinker | — | — | — |
| MAP |  | — | — | — |
| MAI |  | 0.071(0.002)^b^ | — | — |
| AHI |  | — | 0.100(0.009)^a^ | 0.081(0.003)^b^ |

**Notes:** Data are presented as β (SE[β]). Model 1 adjusted for age, body mass index (BMI), sex and plus Smoking status, Alcohol consumption, mean artierial pressure in model 2 and plus MAI in model 3. ^a^p<0.001, ^b^p<0.01, ^c^p<0.05.

**Abbreviations:** BMI, body mass index; AHI, apnea-hyponea index; MAP, mean artierial pressure; MAI, microarousal index; FBG, fasting blood glucose; FIN, fasting insulin; HOMA-IR, homeostasis model assessment of insulin resistance.

**Table S12** Stepwise multiple linear regression **(CT90)** for glucose metabolism index in model 1, 2 and 3 **in Male subjects**

| Variable | Reference | FBG, mmol/L | FIN,$u\text{U}/\text{mL}$ | HOMA-IR |
| --- | --- | --- | --- | --- |
| Model 1 | | | | |
| Age, y |  | 0.201(0.002)^a^ | -0.081(0.016)^a^ | — |
| BMI |  | 0.191(0.009)^a^ | 0.429(0.056)^b^ | 0.405(0.018)^a^ |
| CT90 |  | 0.072(0.004)^b^ | — | — |
| Model 2 | | | | |
| Age, y |  | 0.198(0.002)^a^ | -0.098(0.016)^a^ |  |
| BMI |  | 0.190(0.009)^a^ | 0.431(0.057)^a^ | 0.421(0.017)^a^ |
| Smoking status | non-current smoker | — | — | — |
| Alcohol consumption | non-current drinker | — | — | — |
| MAP |  | — | — | — |
| CT90 |  | 0.071(0.004)^c^ | — | — |
| Model 3 |  |  |  |  |
| Age, y |  | 0.197(0.002)^a^ | -0.094(0.016)^a^ | — |
| BMI |  | 0.188(0.009)^a^ | 0.432(0.056)^a^ | 0.423(0.017)^a^ |
| Smoking status | non-current smoker | — | — | — |
| Alcohol consumption | non-current drinker | — | — | — |
| MAP |  | — | — | — |
| MAI |  | — | — | — |
| CT90 |  | 0.074(0.004)^b^ | — | — |

**Notes:** Data are presented as β (SE[β]). Model 1 adjusted for age, body mass index (BMI), sex and plus Smoking status, Alcohol consumption, mean artierial pressure in model 2 and plus MAI in model 3. ^a^p<0.001, ^b^p<0.01, ^c^p<0.05.

**Abbreviations:** BMI, body mass index; CT90, percentage of total sleep time with SpO2<90%; MAP, mean artierial pressure; MAI, microarousal index; FBG, fasting blood glucose; FIN, fasting insulin; HOMA-IR, homeostasis model assessment of insulin resistance.

**Table S13** Stepwise multiple linear regression **(SASHB)** for glucose metabolism index in model 1, 2 and 3 **in Male subjects**

| Variable | Reference | TC, mmol/L | TG, mmol/L | HDL-C, mmol/L | LDL-C, mmol/L | apoA-I, g/L | apoB, g/L | apoE, mg/dL |
| --- | --- | --- | --- | --- | --- | --- | --- | --- |
| Model 1 | | | | | | | | |
| Age, y |  | — | — | — | — | 0.082(0.002)^b^ | — | -0.059(0.004)^c^ |
| BMI |  | 0.104(0.008)^a^ | 0.166(0.016)^a^ | -0.218(0.002)^a^ | 0.087(0.007)^b^ | -0.101(0.001)^a^ | 0.170(0.002)^a^ | 0.158(0.016)^a^ |
| SASHB |  | 0.094(0.002)^b^ | — | — | 0.066(0.001)^c^ | — | 0.117(0.001)^a^ | 0.076(0.002)^b^ |
| Model 2 | | | | | | | | |
| Age,y |  | — | — | — | — | 0.095(0.001)^b^ |  | -0.059(0.005)^c^ |
| BMI |  | 0.166(0.017)^a^ | 0.166(0.017)^a^ | -0.217(0.002)^a^ | 0.090(0.007)^b^ | -0.105(0.001)^a^ | 0.177(0.002)^a^ | 0.162(0.017)^a^ |
| Smoking status | non-current smoker | — | — | 0.069(0.114)^c^ | — | — | — | — |
| Alcohol consumption | non-current drinker | — | — | — | — | — | 0.057(0.052)^c^ | — |
| MAP |  | — | — | — | — | — | — | — |
| SASHB |  | 0.069(0.114)^b^ | — | — | 0.078(0.002)^b^ | — | 0.113(0.002)^a^ | 0.068(0.001)^c^ |
| Model 3 | | | | | | | | |
| Age,y |  | — | — | — | — | 0.094(0.002)^b^ | — | -0.057(0.005)^c^ |
| BMI |  | 0.106(0.008)^a^ | 0.166(0.017)^a^ | -0.214(0.002)^a^ | 0.099(0.007)^a^ | -0.103(0.001)^a^ | 0.177(0.002)^a^ | 0.172(0.016)^a^ |
| Smoking status | non-current smoker | — | — | 0.069(0.114)^c^ | — | — | — | — |
| Alcohol consumption | non-current drinker | — | — | — | — | — | 0.057(0.052)^c^ | — |
| MAP |  | — | — | — | — | — | — | — |
| MAI |  | 0.080(0.002)^b^ | — | — | 0.088(0.001)^b^ | — | — | 0.076(0.003)^b^ |
| SASHB |  | 0.074(0.002)^c^ | — | — | — | — | 0.111(0.001)^a^ | — |

**Notes:** Data are presented as β (SE[β]). Model 1 adjusted for age, body mass index (BMI), sex and plus Smoking status, Alcohol consumption, mean artierial pressure in model 2 and plus MAI in model 3. ^a^p<0.001, ^b^p<0.01, ^c^p<0.05.

**Abbreviations:** BMI, body mass index; SASHB, sleep apnea-specific hypoxic burden; MAP, mean artierial pressure; MAI, microarousal index;

TC, Total cholesterol; TG, Total triglycerides; HDL-C, High-density lipoprotein cholesterol; LDL-C, Low-density lipoprotein cholesterol; apoA-I, apolipoprotein A-I; apoB, apolipoprotein B; apoE, apolipoprotein E.

**Table S14** Stepwise multiple linear regression **(AHI)** for glucose metabolism index in model 1, 2 and 3 **in Male subjects**

| Variable | Reference | TC, mmol/L | TG, mmol/L | HDL-C, mmol/L | LDL-C, mmol/L | apoA-I, g/L | apoB, g/L | apoE, mg/dL |
| --- | --- | --- | --- | --- | --- | --- | --- | --- |
| Model 1 | | | | | | | | |
| Age, y |  | — | — | — | — | 0.082(0.001)^b^ | — | -0.066(0.004)^c^ |
| BMI |  | 0.091(0.008)^b^ | 0.144(0.017)^a^ | -0.218(0.002)^a^ | 0.076(0.007)^b^ | -0.101(0.001)^a^ | 0.152(0.002)^a^ | 0.140(0.016)^a^ |
| AHI |  | 0.123(0.001)^a^ | 0.076(0.003)^b^ | — | 0.092(0.001)^b^ | — | 0.159(0.002)^a^ | 0.123(0.003)^a^ |
| Model 2 | | | | | | | | |
| Age,y |  | — | — | — | — | 0.095(0.002)^b^ |  | -0.067(0.005)^c^ |
| BMI |  | 0.094(0.008)^b^ | 0.143(0.018)^a^ | -0.217(0.002)^a^ | 0.078(0.007)^b^ | -0.105(0.001)^a^ | 0.163(0.002)^a^ | 0.143(0.017)^a^ |
| Smoking status | non-current smoker | — | — | 0.069(0.114)^c^ | — | — | — | — |
| Alcohol consumption | non-current drinker | — | — | — | — | — | — | — |
| MAP |  | — | — | — | — | — | — | — |
| AHI |  | 0.129(0.001)^a^ | 0.071(0.003)^c^ |  | 0.102(0.001)^b^ |  | 0.158(0.001)^a^ | 0.118(0.003)^a^ |
| Model 3 | | | | | | | | |
| Age,y |  | — | — | — | — | 0.094(0.002)^b^ | — | -0.068(0.005)^c^ |
| BMI |  | 0.095(0.008)^b^ | 0.144(0.018)^a^ | -0.214(0.002)^a^ | 0.078(0.007)^b^ | -0.103(0.001)^a^ | 0.162(0.002)^a^ | 0.143(0.017)^a^ |
| Smoking status | non-current smoker | — | — | 0.069(0.114)^c^ | — | — | — | — |
| Alcohol consumption | non-current drinker | — | — | — | — | — | — | — |
| MAP |  | — | — | — | — | — | — | — |
| MAI |  | 0.064(0.002)^c^ | — | — | — | — | — | — |
| AHI |  | 0.103(0.001)^b^ | 0.071(0.003)^c^ | — | 0.101(0.001)^b^ | — | 0.158(0.001)^a^ | 0.119(0.003)^a^ |

**Notes:** Data are presented as β (SE[β]). Model 1 adjusted for age, body mass index (BMI), sex and plus Smoking status, Alcohol consumption, mean artierial pressure in model 2 and plus MAI in model 3. ^a^p<0.001, ^b^p<0.01, ^c^p<0.05.

**Abbreviations:** BMI, body mass index; AHI, apnea-hyponea index; MAP, mean artierial pressure; MAI, microarousal index;

TC, Total cholesterol; TG, Total triglycerides; HDL-C, High-density lipoprotein cholesterol; LDL-C, Low-density lipoprotein cholesterol; apoA-I, apolipoprotein A-I; apoB, apolipoprotein B; apoE, apolipoprotein E.

**Table S15** Stepwise multiple linear regression **(CT90)** for glucose metabolism index in model 1, 2 and 3 **in Male subjects**

| Variable | Reference | TC, mmol/L | TG, mmol/L | HDL-C, mmol/L | LDL-C, mmol/L | apoA-I, g/L | apoB, g/L | apoE, mg/dL |
| --- | --- | --- | --- | --- | --- | --- | --- | --- |
| Model 1 | | | | | | | | |
| Age, y |  | — | — | — | — | 0.082(0.002)^b^ | — | -0.053(0.004)^c^ |
| BMI |  | 0.112(0.008)^a^ | 0.166(0.016)^a^ | -0.218(0.002)^a^ | 0.084(0.007)^b^ | -0.101(0.001)^a^ | 0.178(0.002)^a^ | 0.162(0.016)^a^ |
| CT90 |  | 0.057(0.004)^c^ | — | — | 0.074(0.003)^b^ | — | 0.083(0.001)^b^ | 0.057(0.007)^c^ |
| Model 2 | | | | | | | | |
| Age,y |  | — | — | — | — | 0.095(0.001)^b^ | — | — |
| BMI |  | 0.118(0.008)^a^ | 0.166(0.017)^a^ | -0.217(0.002)^a^ | 0.087(0.007)^b^ | -0.105(0.001)^a^ | 0.184(0.002)^a^ | 0.184(0.016)^a^ |
| Smoking status | non-current smoker | — | — | 0.069(0.114)^c^ | — | — |  | — |
| Alcohol consumption | non-current drinker | — | — | — | — | — | 0.062(0.052)^c^ | — |
| MAP |  | — | — | — | — | — | — | — |
| CT90 |  | 0.063(0.004)^c^ | — | — | 0.086(0.003)^b^ |  | 0.085(0.001)^b^ | — |
| Model 3 | | | | | | | | |
| Age,y |  | — | — | — | — | 0.094(0.001)^b^ | — | -0.057(0.005)^c^ |
| BMI |  | 0.123(0.008)^a^ | 0.166(0.017)^a^ | -0.214(0.002)^a^ | 0.085(0.007)^b^ | -0.103(0.001)^a^ | 0.181(0.002)^a^ | 0.172(0.016)^a^ |
| Smoking status | non-current smoker | — | — | 0.069(0.114)^c^ | — | — | — | — |
| Alcohol consumption | non-current drinker | — | — | — | — | — | — | — |
| MAP |  | — | — | — | — | — | — | — |
| MAI |  | 0.102(0.002)^a^ | — | — | 0.068(0.001)^c^ | — | 0.062(0.002)^c^ | 0.076(0.003)^b^ |
| CT90 |  | — | — | — | 0.061(0.003)^c^ | — | 0.062(0.001)^c^ | — |

**Notes:** Data are presented as β (SE[β]). Model 1 adjusted for age, body mass index (BMI), sex and plus Smoking status, Alcohol consumption, mean artierial pressure in model 2 and plus MAI in model 3. ^a^p<0.001, ^b^p<0.01, ^c^p<0.05.

**Abbreviations:** BMI, body mass index; CT90, percentage of total sleep time with SpO_2_<90%; MAP, mean artierial pressure; MAI, microarousal index;

TC, Total cholesterol; TG, Total triglycerides; HDL-C, High-density lipoprotein cholesterol; LDL-C, Low-density lipoprotein cholesterol; apoA-I, apolipoprotein A-I; apoB, apolipoprotein B; apoE, apolipoprotein E.

**Table S16** Adjusted odds ratios for abnormal glucose and lipid metabolism according to SASHB categories in models 1, 2 and 3 **in Male subjects**

|  | Hyperglycemia | Hyperinsulinemia | HOMA-IR≥2.5 | Hyper-total cholesterolemia | Hyper-LDL cholesterolemia | Hypo-HDL cholesterolemia | Hyper- triglyceridemia |
| --- | --- | --- | --- | --- | --- | --- | --- |
| Adjusted OR(95% CI) in model 1 | | | | | | | |
| SASHB≤25.64 | 1 | 1 | 1 | 1 | 1 | 1 | 1 |
| 25.64 < SASHB≤95.73 | 1.318(0.893,1.945) | 1.240(0.867,1.773) | 1.558(1.104,2.199) | 1.506(1.039,2.181) | 1.545(1.050,2.272) | 0.968(0.708,1.323) | 1.541(1.119,2.121) |
| 95.73< SASHB≤238.80 | 0.909(0.611,1.352) | 1.136(0.794,1.626) | 1.121(0.791,1.588) | 1.605(1.107,2.327) | 1.535(1.039,2.267) | 0.815(0.593,1.122) | 1.489(1.077,2.058) |
| SASHB>238.80 | 1.386(0.942,2.039) | 1.611(1.125,2.307) | 1.785(1.251,2.547) | 2.007(1.386,2.906) | 1.881(1.277,2.771) | 0.910(0.655,1.265) | 1.863(1.340,2.589) |
| P-value for linear trend | 0.045^*^ | 0.021^*^ | 0.006^*^ | 0.002^*^ | 0.026^*^ | 0.590 | 0.006^*^ |
| Adjusted OR(95% CI) in model 2 | | | | | | | |
| SASHB≤25.64 | 1 | 1 | 1 | 1 | 1 | 1 | 1 |
| 25.64 < SASHB≤95.73 | 1.293(0.856,1.953) | 1.321(0.905,1.929) | 1.667(1.159,2.397) | 1.551(1.042,2.309) | 1.709(1.125,2.596) | 0.985(0.709,1.368) | 1.502(1.073,2.102) |
| 95.73< SASHB≤238.80 | 0.915(0.599,1.398) | 1.228(0.837,1.802) | 1.184(0.817,1.715) | 1.708(1.143,2.553) | 1.683(1.097,2.582) | 0.801(0.571,1.123) | 1.420(1.006,2.004) |
| SASHB>238.80 | 1.316(0.869,1.993) | 1.752(1.195,2.569) | 1.857(1.274,2.707) | 2.228(1.495,3.321) | 2.264(1.484,3.453) | 0.905(0.638,1.283) | 1.680(1.184,2.384) |
| P-value for linear trend | 0.097 | 0.009^*^ | 0.005^*^ | ＜0.001^*^ | 0.005^*^ | 0.610 | 0.055 |
| Adjusted OR(95% CI) in model 3 | | | | | | | |
| SASHB≤25.64 | 1 | 1 | 1 | 1 | 1 | 1 | 1 |
| 25.64 < SASHB≤95.73 | 1.315(0.869,1.99) | 1.317(0.902,1.922) | 1.691(1.175,2.435) | 1.515(1.017,2.258) | 1.681(1.106,2.555) | 1.000(0.719,1.389) | 1.517(1.083,2.126) |
| 95.73< SASHB≤238.80 | 0.909(0.592,1.395) | 1.221(0.830,1.797) | 1.193(0.820,1.736) | 1.588(1.058,2.385) | 1.606(1.042,2.475) | 0.824(0.585,1.160) | 1.429(1.008,2.027) |
| SASHB>238.80 | 1.266(0.825,1.943) | 1.719(1.158,2.551) | 1.845(1.249,2.723) | 1.974(1.308,2.981) | 2.097(1.356,3.244) | 0.935(0.651,1.342) | 1.681(1.170,2.417) |
| P-value for linear trend | 0.157 | 0.018^*^ | 0.009^*^ | 0.008^*^ | 0.026^*^ | 0.730 | 0.084 |

**Notes:** ORs were adjusted for age, body mass index (BMI), sex and plus Smoking status, Alcohol consumption, mean artierial pressure in model 2 and plus MAI in model 3; P-values for linear trends were determined by examining the median SASHB value for each quartile and assessing the overall F test for the median MAI variable. *P<0.05.

**Abbreviations:** SASHB, sleep apnea-specific hypoxic burden.

**Table S17** Adjusted odds ratios for abnormal glucose and lipid metabolism according to AHI categories in models 1, 2 and 3 **in Male subjects**

|  | Hyperglycemia | Hyperinsulinemia | HOMA-IR≥2.5 | Hyper-total cholesterolemia | Hyper-LDL cholesterolemia | Hypo-HDL cholesterolemia | Hyper- triglyceridemia |
| --- | --- | --- | --- | --- | --- | --- | --- |
| Adjusted OR(95% CI) in model 1 | | | | | | | |
| AHI< 5 | 1 | 1 | 1 | 1 | 1 | 1 | 1 |
| 5≤AHI< 15 | 0.858(0.553,1.333) | 1.576(1.060,2.342) | 1.297(0.889,1.893) | 1.588(1.036,2.433) | 1.936(1.243,3.014) | 0.947(0.673,1.333) | 1.572(1.095,2.258) |
| 15≤AHI  ＜30 | 0.875(0.574,1.332) | 1.290(0.871,1.911) | 1.079(0.74,1.574) | 2.373(1.578,3.569) | 2.371(1.540,3.651) | 0.787(0.558,1.110) | 2.572(1.803,3.670) |
| AHI≥30 | 1.201(0.837,1.722) | 1.613(1.138,2.286) | 1.626(1.163,2.273) | 2.427(1.674,3.519) | 2.318(1.561,3.442) | 0.947(0.696,1.289) | 2.595(1.887,3.569) |
| P-value for linear trend | 0.031^*^ | 0.043^*^ | 0.001^*^ | ＜0.001^*^ | 0.004^*^ | 0.840 | ＜0.001^*^ |
| Adjusted OR(95% CI) in model 2 | | | | | | | |
| AHI≤5 | 1 | 1 | 1 | 1 | 1 | 1 | 1 |
| 5 < AHI≤15 | 0.799(0.501,1.276) | 1.801(1.177,2.756) | 1.405(0.939,2.103) | 1.595(1.003,2.535) | 2.215(1.362,3.603) | 0.859(0.598,1.233) | 1.430(0.975,2.097) |
| 15< AHI≤30 | 0.863(0.552,1.349) | 1.467(0.961,2.237) | 1.167(0.781,1.744) | 2.533(1.631,3.933) | 2.706(1.685,4.343) | 0.750(0.521,1.079) | 2.503(1.719,3.645) |
| AHI>30 | 1.152(0.781,1.700) | 1.933(1.325,2.820) | 1.807(1.261,2.588) | 2.686(1.792,4.028) | 2.802(1.805,4.350) | 0.891(0.641,1.240) | 2.359(1.680,3.313) |
| P-value for linear trend | 0.080 | 0.011^*^ | 0.001^*^ | ＜0.001^*^ | 0.001^*^ | 0.902 | ＜0.001^*^ |
| Adjusted OR(95% CI) in model 3 | | | | | | | |
| AHI≤5 | 1 | 1 | 1 | 1 | 1 | 1 | 1 |
| 5 < AHI≤15 | 0.794(0.497,1.268) | 1.797(1.174,2.751) | 1.405(0.939,2.105) | 1.564(0.983,2.488) | 2.190(1.346,3.564) | 0.866(0.603,1.245) | 1.441(0.982,2.114) |
| 15< AHI≤30 | 0.831(0.529,1.304) | 1.461(0.955,2.236) | 1.163(0.776,1.744) | 2.430(1.560,3.785) | 2.644(1.642,4.259) | 0.761(0.527,1.098) | 2.568(1.757,3.752) |
| AHI>30 | 1.102(0.736,1.649) | 1.925(1.301,2.847) | 1.786(1.229,2.595) | 2.425(1.597,3.684) | 2.633(1.672,4.145) | 0.921(0.653,1.299) | 2.403(1.687,3.424) |
| P-value for linear trend | 0.156 | 0.018^*^ | 0.001^*^ | 0.001^*^ | 0.011^*^ | 0.863 | ＜0.001^*^ |

**Notes:** ORs were adjusted for age, body mass index (BMI), sex and plus Smoking status, Alcohol consumption, mean artierial pressure in model 2 and plus MAI in model 3; P-values for linear trends were determined by examining the median AHI value for each quartile and assessing the overall F test for the median MAI variable. *P<0.05.

**Abbreviations:** AHI, apnea-hyponea index.

**Table S18** Adjusted odds ratios for abnormal glucose and lipid metabolism according to CT90 categories in models 1, 2 and 3 **in Male subjects**

|  | Hyperglycemia | Hyperinsulinemia | HOMA-IR≥2.5 | Hyper-total cholesterolemia | Hyper-LDL cholesterolemia | Hypo-HDL cholesterolemia | Hyper- triglyceridemia |
| --- | --- | --- | --- | --- | --- | --- | --- |
| Adjusted OR(95% CI) in model 1 | | | | | | | |
| CT90≤0.18 | 1 | 1 | 1 | 1 | 1 | 1 | 1 |
| 0.18< CT90≤3.73 | 0.995(0.714,1.385) | 1.297(0.957,1.759) | 1.180(0.880,1.582) | 1.522(1.115,2.078) | 1.677(1.212,2.320) | 0.832(0.635,1.091) | 1.564(1.191,2.053) |
| CT90＞3.73 | 1.314(0.950,1.819) | 1.535(1.130,2.087) | 1.489(1.101,2.013) | 1.849(1.354,2.524) | 1.759(1.266,2.444) | 0.857(0.645,1.138) | 1.551(1.172,2.052) |
| P-value for linear trend | 0.009^*^ | 0.224 | 0.148 | 0.196 | 0.041^*^ | 0.136 | 0.156 |
| Adjusted OR(95% CI) in model 2 | | | | | | | |
| CT90≤0.18 | 1 | 1 | 1 | 1 | 1 | 1 | 1 |
| 0.18< CT90≤3.73 | 0.984(0.696,1.392) | 1.351(0.980,1.861) | 1.236(0.908,1.681) | 1.572(1.130,2.188) | 1.721(1.218,2.432) | 0.842(0.634,1.118) | 1.467(1.102,1.952) |
| CT90＞3.73 | 1.266(0.898,1.786) | 1.658(1.199,2.292) | 1.555(1.131,2.139) | 2.036(1.461,2.836) | 1.982(1.396,2.814) | 0.832(0.616,1.123) | 1.510(1.123,2.032) |
| P-value for linear trend | 0.018^*^ | 0.107 | 0.103 | 0.086 | 0.012^*^ | 0.211 | 0.258 |
| Adjusted OR(95% CI) in model 3 | | | | | | | |
| CT90≤0.18 | 1 | 1 | 1 | 1 | 1 | 1 | 1 |
| 0.18< CT90≤3.73 | 0.951(0.670,1.349) | 1.348(0.977,1.859) | 1.218(0.894,1.660) | 1.526(1.094,2.128) | 1.693(1.196,2.398) | 0.844(0.634,1.122) | 1.463(1.097,1.951) |
| CT90＞3.73 | 1.224(0.856,1.750) | 1.644(1.175,2.299) | 1.532(1.101,2.131) | 1.828(1.296,2.579) | 1.850(1.286,2.663) | 0.851(0.623,1.161) | 1.483(1.090,2.019) |
| P-value for linear trend | 0.028^*^ | 0.188 | 0.179 | 0.514 | 0.066 | 0.263 | 0.458 |

**Notes:** ORs were adjusted for age, body mass index (BMI), sex and plus Smoking status, Alcohol consumption, mean artierial pressure in model 2 and plus MAI in model 3; P-values for linear trends were determined by examining the median CT90 value for each quartile and assessing the overall F test for the median MAI variable. *P<0.05.

**Abbreviations:** CT90, percentage of total sleep time with SpO_2_<90%.

**Table S19** Stepwise multiple linear regression **(SASHB)** for glucose metabolism index in model 1, 2 and 3 **in Female subjects**

| Variable | Reference | FBG, mmol/L | FIN,$u\text{U}/\text{mL}$ | HOMA-IR |
| --- | --- | --- | --- | --- |
| Model 1 | | | | |
| Age, y |  | 0.128(0.005)^b^ | — | — |
| BMI |  | 0.305(0.013)^a^ | 0.506(0.063)^a^ | 0.455(0.024)^a^ |
| SASHB |  | 0.103(0.001)^c^ | 0.150(0.002)^b^ | 0.125(0.001)^b^ |
| Model 2 | | | | |
| Age, y |  | 0.129(0.005)^b^ | — | — |
| BMI |  | 0.327(0.015)^a^ | 0.518(0.067)^a^ | 0.472(0.026)^a^ |
| Smoking status | non-current smoker | — | — | — |
| Alcohol consumption | non-current drinker | — | 0.080(1.417)^c^ | — |
| MAP |  | — | — | — |
| SASHB |  | 0.109(0.001)^c^ | 0.150(0.003)^b^ | 0.126(0.001)^b^ |
| Model 3 |  |  |  |  |
| Age, y |  | 0.130(0.005)^b^ | — | — |
| BMI |  | 0.328(0.015)^a^ | 0.518(0.067)^a^ | 0.473(0.026)^a^ |
| Smoking status | non-current smoker | — | — | — |
| Alcohol consumption | non-current drinker | — | — | — |
| MAP |  | — | — | — |
| MAI |  | — | — | — |
| SASHB |  | 0.112(0.001)^c^ | 0.151(0.003)^b^ | 0.132(0.001)^b^ |

**Notes:** Data are presented as β (SE[β]). Model 1 adjusted for age, body mass index (BMI), sex and plus Smoking status, Alcohol consumption, mean artierial pressure in model 2 and plus MAI in model 3. ^a^p<0.001, ^b^p<0.01, ^c^p<0.05.

**Abbreviations:** BMI, body mass index; SASHB, sleep apnea-specific hypoxic burden; MAP, mean artierial pressure; MAI, microarousal index; FBG, fasting blood glucose; FIN, fasting insulin; HOMA-IR, homeostasis model assessment of insulin resistance.

**Table S20** Stepwise multiple linear regression **(AHI)** for glucose metabolism index in model 1, 2 and 3 **in Female subjects**

| Variable | Reference | FBG, mmol/L | FIN,$u\text{U}/\text{mL}$ | HOMA-IR |
| --- | --- | --- | --- | --- |
| Model 1 | | | | |
| Age, y |  | 0.107(0.005)c | — | — |
| BMI |  | 0.280(0.014)a | 0.492(0.065)a | 0.436(0.025)a |
| AHI |  | 0.161(0.003)b | 0.154(0.016)b | 0.153(0.006)b |
| Model 2 | | | | |
| Age, y |  | 0.108(0.005)c | — | — |
| BMI |  | 0.301(0.015)a | 0.502(0.069)a | 0.45(0.027)a |
| Smoking status | non-current smoker | — | — | — |
| Alcohol consumption | non-current drinker | — | — | — |
| MAP |  | — | — | — |
| AHI |  | 0.173(0.004)b | 0.169(0.017)a | 0.170(0.006)b |
| Model 3 |  |  |  |  |
| Age, y |  | 0.109(0.005)c | — | — |
| BMI |  | 0.302(0.015)a | 0.502(0.069)a | 0.451(0.027)a |
| Smoking status | non-current smoker | — | — | — |
| Alcohol consumption | non-current drinker | — | — | — |
| MAP |  | — | — | — |
| MAI |  | — | — | — |
| AHI |  | 0.184(0.004)b | 0.174(0.018)a | 0.184(0.007)a |

**Notes:** Data are presented as β (SE[β]). Model 1 adjusted for age, body mass index (BMI), sex and plus Smoking status, Alcohol consumption, mean artierial pressure in model 2 and plus MAI in model 3. ^a^p<0.001, ^b^p<0.01, ^c^p<0.05.

**Abbreviations:** BMI, body mass index; AHI, apnea-hyponea index; MAP, mean artierial pressure; MAI, microarousal index; FBG, fasting blood glucose; FIN, fasting insulin; HOMA-IR, homeostasis model assessment of insulin resistance.

**Table S21** Stepwise multiple linear regression **(CT90)** for glucose metabolism index in model 1, 2 and 3 **in Female subjects**

| Variable | Reference | FBG, mmol/L | FIN,$u\text{U}/\text{mL}$ | HOMA-IR |
| --- | --- | --- | --- | --- |
| Model 1 | | | | |
| Age, y |  | 0.149(0.005)^b^ | — | — |
| BMI |  | 0.311(0.014)^a^ | 0.520(0.064)^a^ | 0.464(0.024)^a^ |
| CT90 |  | — | — | — |
| Model 2 | | | | |
| Age, y |  | 0.154(0.005)^b^ | — | — |
| BMI |  | 0.339(0.015)^a^ | 0.533(0.068)^a^ | 0.483(0.026)^a^ |
| Smoking status | non-current smoker | — | — | — |
| Alcohol consumption | non-current drinker | — | — | — |
| MAP |  | — | — | — |
| CT90 |  | — | — | — |
| Model 3 |  |  |  |  |
| Age, y |  | 0.154(0.005)^b^ | — | — |
| BMI |  | 0.339(0.015)^a^ | 0.533(0.068)^a^ | 0.484(0.026)^a^ |
| Smoking status | non-current smoker | — | — | — |
| Alcohol consumption | non-current drinker | — | 0.094(1.427)^c^ | — |
| MAP |  | — | — | — |
| MAI |  | — | — | — |
| CT90 |  | — | — | — |

**Notes:** Data are presented as β (SE[β]). Model 1 adjusted for age, body mass index (BMI), sex and plus Smoking status, Alcohol consumption, mean artierial pressure in model 2 and plus MAI in model 3. ^a^p<0.001, ^b^p<0.01, ^c^p<0.05.

**Abbreviations:** BMI, body mass index; CT90, percentage of total sleep time with SpO_2_<90%; MAP, mean artierial pressure; MAI, microarousal index; FBG, fasting blood glucose; FIN, fasting insulin; HOMA-IR, homeostasis model assessment of insulin resistance.

**Table S22** Stepwise multiple linear regression **(SASHB)** for glucose metabolism index in model 1, 2 and 3 **in Female subjects**

| Variable | Reference | TC, mmol/L | TG, mmol/L | HDL-C, mmol/L | LDL-C, mmol/L | apoA-I, g/L | apoB, g/L | apoE, mg/dL |
| --- | --- | --- | --- | --- | --- | --- | --- | --- |
| Model 1 | | | | | | | | |
| Age, y |  | 0.404(0.003)^a^ | 0.152(0.007)^b^ | 0.140(0.001)^b^ | 0.288(0.003)^a^ | 0.274(0.001)^a^ | 0.355(0.001)^a^ | 0.128(0.008)^c^ |
| BMI |  | — | 0.165(0.018)^a^ | -0.269(0.003)^a^ | — | -0.169(0.002)^a^ | 0.161(0.002)^a^ | 0.128(0.023)^b^ |
| SASHB |  | — | 0.146(0.001)^b^ | -0.128(0.002)^c^ | — | — | 0.149(0.002)^b^ | 0.125(0.001)^c^ |
| Model 2 | | | | | | | | |
| Age,y |  | 0.393(0.004)^a^ | 0.159(0.006)^b^ | 0.133(0.001)^b^ | 0.284(0.003)^a^ | 0.268(0.001)^a^ | 0.350(0.001)^a^ | 0.133(0.008)^b^ |
| BMI |  | — | 0.165(0.017)^b^ | -0.257(0.003)^a^ | 0.100(0.008)^c^ | -0.169(0.002)^b^ | 0.149(0.002)^b^ | 0.111(0.023)^c^ |
| Smoking status | non-current smoker | — | — | — | — | — | — | — |
| Alcohol consumption | non-current drinker | — | — | — | — | — | — | — |
| MAP |  | — | — | — | -0.118(0.108)^c^ | — | — | — |
| SASHB |  | — | 0.169(0.001)^b^ | -0.128(0.001)^c^ | — | — | 0.144(0.001)^b^ | 0.139(0.001)^c^ |
| Model 3 | | | | | | | | |
| Age,y |  | 0.397(0.004)^a^ | 0.158(0.006)^b^ | 0.131(0.001)^b^ | 0.288(0.003)^a^ | 0.271(0.001)^a^ | 0.353(0.001)^a^ | 0.135(0.008)^b^ |
| BMI |  | — | 0.163(0.017)^b^ | -0.259(0.003)^a^ | 0.106(0.008)^c^ | -0.163(0.002)^b^ | 0.156(0.002)^b^ | 0.114(0.023)^c^ |
| Smoking status | non-current smoker | — | — | — | — | — | — | — |
| Alcohol consumption | non-current drinker | — | — | — | — | — | — | — |
| MAP |  | — | — | — | -0.132(0.109)^b^ | — | — | — |
| MAI |  | — | — | — | — | — | — | — |
| SASHB |  | — | 0.162(0.001)^b^ | -0.138(0.002)^c^ | — | — | 0.165(0.002)^b^ | 0.148(0.001)^b^ |

**Notes:** Data are presented as β (SE[β]). Model 1 adjusted for age, body mass index (BMI), sex and plus Smoking status, Alcohol consumption, mean artierial pressure in model 2 and plus MAI in model 3. ^a^p<0.001, ^b^p<0.01, ^c^p<0.05.

**Abbreviations:** BMI, body mass index; SASHB, sleep apnea-specific hypoxic burden; MAP, mean artierial pressure; MAI, microarousal index;

TC, Total cholesterol; TG, Total triglycerides; HDL-C, High-density lipoprotein cholesterol; LDL-C, Low-density lipoprotein cholesterol; apoA-I, apolipoprotein A-I; apoB, apolipoprotein B; apoE, apolipoprotein E.

**Table S23** Stepwise multiple linear regression **(AHI)** for glucose metabolism index in model 1, 2 and 3 **in Female subjects**

| Variable | Reference | TC, mmol/L | TG, mmol/L | HDL-C, mmol/L | LDL-C, mmol/L | apoA-I, g/L | apoB, g/L | apoE, mg/dL |
| --- | --- | --- | --- | --- | --- | --- | --- | --- |
| Model 1 | | | | | | | | |
| Age, y |  | 0.383(0.003)^a^ | 0.125(0.007)^c^ | 0.136(0.001)^b^ | 0.280(0.003)^a^ | 0.273(0.001)^a^ | 0.354(0.001)^a^ | 0.111(0.008)^c^ |
| BMI |  | — | 0.131(0.018)^b^ | -0.262(0.003)^a^ | — | -0.168(0.002)^b^ | 0.149(0.002)^b^ | 0.103(0.023)^c^ |
| AHI |  | 0.105(0.002)^c^ | 0.224(0.005)^a^ | -0.115(0.001)^c^ | — | — | 0.148(0.001)^b^ | 0.176(0.006)^b^ |
| Model 2 | | | | | | | | |
| Age,y |  | 0.376(0.004)^a^ | 0.139(0.006)^b^ | 0.130(0.001)^c^ | 0.274(0.003)^a^ | 0.266(0.001)^a^ | 0.351(0.001)^a^ | 0.123(0.008)^c^ |
| BMI |  | — | 0.136(0.017)^b^ | -0.252(0.003)^a^ | — | -0.170(0.002)^b^ | 0.142(0.002)^b^ | — |
| Smoking status | non-current smoker | — | — | — | — | — | — | — |
| Alcohol consumption | non-current drinker | — | — | — | — | — | — | — |
| MAP |  | — | — | — | -0.112(0.108)^c^ | — | — | 0.102(0.278)^c^ |
| AHI |  | — | 0.229(0.004)^a^ | -0.116(0.001)^c^ | — | — | 0.137(0.001)^b^ | 0.169(0.006)^b^ |
| Model 3 | | | | | | | | |
| Age,y |  | 0.379(0.004)^a^ | 0.138(0.006)^b^ | 0.129(0.001)^c^ | 0.276(0.003)^a^ | 0.268(0.001)^a^ | 0.352(0.001)^a^ | 0.123(0.008)^c^ |
| BMI |  | — | 0.136(0.017)^b^ | -0.253(0.003)^a^ | — | -0.167(0.002)^b^ | 0.145(0.002)^b^ | — |
| Smoking status | non-current smoker | — | — | — | — | — | — | — |
| Alcohol consumption | non-current drinker | — | — | — | — | — | — | — |
| MAP |  | — | — | — | -0.125(0.109)^c^ | — | — | — |
| MAI |  | -0.111(0.002)^c^ | — | — | — | — | -0.098(0.001)^c^ | — |
| AHI |  | 0.129(0.003)^c^ | 0.227(0.004)^a^ | -0.129(0.001)^c^ | — | — | 0.168(0.001)^b^ | 0.185(0.006)^b^ |

**Notes:** Data are presented as β (SE[β]). Model 1 adjusted for age, body mass index (BMI), sex and plus Smoking status, Alcohol consumption, mean artierial pressure in model 2 and plus MAI in model 3. ^a^p<0.001, ^b^p<0.01, ^c^p<0.05.

**Abbreviations:** BMI, body mass index; AHI, apnea-hyponea index; MAP, mean artierial pressure; MAI, microarousal index;

TC, Total cholesterol; TG, Total triglycerides; HDL-C, High-density lipoprotein cholesterol; LDL-C, Low-density lipoprotein cholesterol; apoA-I, apolipoprotein A-I; apoB, apolipoprotein B; apoE, apolipoprotein E.

**Table S24** Stepwise multiple linear regression **(CT90)** for glucose metabolism index in model 1, 2 and 3 **in Female subjects**

| Variable | Reference | TC, mmol/L | TG, mmol/L | HDL-C, mmol/L | LDL-C, mmol/L | apoA-I, g/L | apoB, g/L | apoE, mg/dL |
| --- | --- | --- | --- | --- | --- | --- | --- | --- |
| Model 1 | | | | | | | | |
| Age, y |  | 0.418(0.003)^a^ | 0.181(0.007)^a^ | 0.108(0.001)^c^ | 0.296(0.003)^a^ | 0.250(0.001)^a^ | 0.385(0.001)^a^ | 0.150(0.008)^b^ |
| BMI |  | — | 0.172(0.018)^a^ | -0.283(0.003)^a^ | — | -0.192(0.002)^a^ | 0.170(0.002)^a^ | 0.129(0.023)^b^ |
| CT90 |  | — | 0.104(0.018)^c^ | — | — | — | 0.099(0.002)^c^ | 0.107(0.022)^c^ |
| Model 2 | | | | | | | | |
| Age,y |  | 0.407(0.003)^a^ | 0.193(0.006)^a^ | 0.102(0.001)^c^ | 0.286(0.003)^a^ | 0.250(0.001)^a^ | 0.378(0.001)^a^ | 0.156(0.008)^b^ |
| BMI |  | — | 0.178(0.017)^a^ | -0.274(0.003)^a^ | 0.109(0.008)^c^ | -0.193(0.002)^a^ | 0.160(0.002)^b^ | 0.116(0.023)^c^ |
| Smoking status | non-current smoker | — | — | — | — | — | — | — |
| Alcohol consumption | non-current drinker | — | — | — | — | — | — | — |
| MAP |  | — | — | — | -0.125(0.107)^c^ | — | — | — |
| CT90 |  | — | 0.112(0.016)^c^ | — | — | — | 0.097(0.002)^c^ | 0.117(0.021)^c^ |
| Model 3 | | | | | | | | |
| Age,y |  | 0.414(0.003)^a^ | 0.190(0.006)^a^ | 0.100(0.001)^c^ | 0.293(0.003)^a^ | 0.258(0.001)^a^ | 0.384(0.001)^a^ | 0.159(0.008)^b^ |
| BMI |  | — | 0.175(0.017)^a^ | -0.276(0.003)^a^ | 0.114(0.008)^c^ | -0.185(0.002)^a^ | 0.166(0.002)^a^ | 0.118(0.023)^c^ |
| Smoking status | non-current smoker | — | — | — | — | — | — | — |
| Alcohol consumption | non-current drinker | — | — | — | — | — | 0.092(0.046)^c^ | — |
| MAP |  | — | — | — | -0.137(0.109)^b^ | — | — | — |
| MAI |  | — | — | — | — | -0.104(0.001)^c^ | — | — |
| CT90 |  | — | 0.103(0.016)^c^ | — | — | 0.103(0.002)^c^ | 0.114(0.002)^c^ | 0.124(0.021)^c^ |

**Notes:** Data are presented as β (SE[β]). Model 1 adjusted for age, body mass index (BMI), sex and plus Smoking status, Alcohol consumption, mean artierial pressure in model 2 and plus MAI in model 3. ^a^p<0.001, ^b^p<0.01, ^c^p<0.05.

**Abbreviations:** BMI, body mass index; AHI, apnea-hyponea index; MAP, mean artierial pressure; MAI, microarousal index;

TC, Total cholesterol; TG, Total triglycerides; HDL-C, High-density lipoprotein cholesterol; LDL-C, Low-density lipoprotein cholesterol; apoA-I, apolipoprotein A-I; apoB, apolipoprotein B; apoE, apolipoprotein E.

**Table S25** Adjusted odds ratios for abnormal glucose and lipid metabolism according to SASHB categories in models 1, 2 and 3 **in Female subjects**

|  | Hyperglycemia | Hyperinsulinemia | HOMA-IR≥2.5 | Hyper-total cholesterolemia | Hyper-LDL cholesterolemia | Hypo-HDL cholesterolemia | Hyper- triglyceridemia |
| --- | --- | --- | --- | --- | --- | --- | --- |
| Adjusted OR(95% CI) in model 1 | | | | | | | |
| SASHB≤10.40 | 1 | 1 | 1 | 1 | 1 | 1 | 1 |
| 10.40 < SASHB≤41.13 | 0.695(0.313,1.542) | 3.307(1.460,7.488) | 1.872(0.920,3.808) | 0.971(0.460,2.049) | 0.855(0.384,1.902) | 1.289(0.695,2.390) | 2.172(1.025,4.603) |
| 41.13< SASHB≤144.88 | 1.287(0.612,2.707) | 4.951(2.112,11.607) | 2.802(1.338,5.871) | 2.42(1.202,4.871) | 1.714(0.818,3.592) | 1.144(0.580,2.256) | 1.459(0.676,3.147) |
| SASHB>144.88 | 1.506(0.710,3.194) | 5.588(2.340,13.341) | 2.696(1.255,5.790) | 1.589(0.767,3.294) | 1.384(0.639,2.996) | 1.938(0.981,3.829) | 3.689(1.733,7.856) |
| P-value for linear trend | 0.005^*^ | 0.006^*^ | 0.089 | 0.389 | 0.393 | 0.404 | 0.008^*^ |
| Adjusted OR(95% CI) in model 2 | | | | | | | |
| SASHB≤10.40 | 1 | 1 | 1 | 1 | 1 | 1 | 1 |
| 10.40 < SASHB≤41.13 | 0.701(0.310,1.586) | 4.678(1.913,11.441) | 2.249(1.072,4.717) | 0.982(0.459,2.103) | 0.803(0.359,1.796) | 1.210(0.649,2.256) | 2.668(1.207,5.894) |
| 41.13< SASHB≤144.88 | 0.988(0.440,2.215) | 6.304(2.430,16.353) | 3.127(1.401,6.980) | 2.416(1.145,5.097) | 1.354(0.623,2.942) | 1.051(0.515,2.144) | 1.761(0.769,4.031) |
| SASHB>144.88 | 1.490(0.666,3.333) | 8.643(3.270,22.845) | 3.417(1.489,7.844) | 1.677(0.765,3.678) | 1.126(0.498,2.542) | 1.930(0.945,3.942) | 4.816(2.111,10.986) |
| P-value for linear trend | 0.003^*^ | 0.002^*^ | 0.045^*^ | 0.532 | 0.772 | 0.470 | 0.002^*^ |
| Adjusted OR(95% CI) in model 3 | | | | | | | |
| SASHB≤10.40 | 1 | 1 | 1 | 1 | 1 | 1 | 1 |
| 10.40 < SASHB≤41.13 | 0.702(0.310,1.588) | 4.691(1.916,11.488) | 2.246(1.071,4.714) | 0.958(0.445,2.064) | 0.794(0.354,1.783) | 1.212(0.650,2.263) | 2.683(1.212,5.939) |
| 41.13< SASHB≤144.88 | 1.000(0.445,2.246) | 6.337(2.433,16.504) | 3.118(1.393,6.978) | 2.615(1.227,5.573) | 1.427(0.651,3.128) | 1.056(0.517,2.160) | 1.731(0.755,3.971) |
| SASHB>144.88 | 1.529(0.680,3.437) | 8.717(3.265,23.273) | 3.4(1.470,7.863) | 1.976(0.888,4.395) | 1.28(0.560,2.923) | 1.947(0.946,4.006) | 4.609(2.012,10.561) |
| P-value for linear trend | 0.002^*^ | 0.002^*^ | 0.051 | 0.147 | 0.342 | 0.473 | 0.005^*^ |

**Notes:** ORs were adjusted for age, body mass index (BMI), sex and plus Smoking status, Alcohol consumption, mean artierial pressure in model 2 and plus MAI in model 3; P-values for linear trends were determined by examining the median SASHB value for each quartile and assessing the overall F test for the median MAI variable. *P<0.05.

**Abbreviations:** SASHB, sleep apnea-specific hypoxic burden.

**Table S26** Adjusted odds ratios for abnormal glucose and lipid metabolism according to AHI categories in models 1, 2 and 3 **in Female subjects**

|  | Hyperglycemia | Hyperinsulinemia | HOMA-IR≥2.5 | Hyper-total cholesterolemia | Hyper-LDL cholesterolemia | Hypo-HDL cholesterolemia | Hyper- triglyceridemia |
| --- | --- | --- | --- | --- | --- | --- | --- |
| Adjusted OR(95% CI) in model 1 | | | | | | | |
| AHI≤5 | 1 | 1 | 1 | 1 | 1 | 1 | 1 |
| 5 < AHI≤15 | 1.837(0.909,3.713) | 1.121(0.551,2.280) | 1.011(0.510,2.004) | 2.612(1.345,5.075) | 2.360(1.161,4.799) | 0.742(0.388,1.422) | 0.913(0.460,1.811) |
| 15< AHI≤30 | 1.497(0.714,3.137) | 1.881(0.932,3.797) | 2.135(1.083,4.206) | 2.249(1.141,4.431) | 2.070(0.993,4.313) | 0.610(0.303,1.227) | 0.890(0.446,1.776) |
| AHI>30 | 2.367(1.208,4.635) | 2.164(1.107,4.228) | 1.935(1.008,3.715) | 2.751(1.447,5.232) | 2.185(1.087,4.393) | 1.359(0.740,2.494) | 2.395(1.296,4.424) |
| P-value for linear trend | 0.023^*^ | 0.012^*^ | 0.033^*^ | 0.033^*^ | 0.229 | 0.305 | 0.009^*^ |
| Adjusted OR(95% CI) in model 2 | | | | | | | |
| AHI≤5 | 1 | 1 | 1 | 1 | 1 | 1 | 1 |
| 5 < AHI≤15 | 1.574(0.748,3.316) | 1.136(0.535,2.410) | 1.070(0.522,2.195) | 2.787(1.405,5.527) | 2.174(1.054,4.482) | 0.596(0.298,1.189) | 0.976(0.480,1.985) |
| 15< AHI≤30 | 1.413(0.624,3.198) | 2.060(0.931,4.555) | 2.480(1.148,5.359) | 2.132(0.994,4.573) | 1.510(0.668,3.409) | 0.600(0.282,1.277) | 1.024(0.477,2.196) |
| AHI>30 | 2.367(1.146,4.889) | 2.732(1.309,5.702) | 2.361(1.148,4.853) | 2.618(1.297,5.284) | 1.845(0.876,3.885) | 1.269(0.663,2.429) | 2.851(1.453,5.592) |
| P-value for linear trend | 0.016^*^ | 0.003^*^ | 0.013^*^ | 0.078 | 0.438 | 0.404 | 0.004^*^ |
| Adjusted OR(95% CI) in model 3 | | | | | | | |
| AHI≤5 | 1 | 1 | 1 | 1 | 1 | 1 | 1 |
| 5 < AHI≤15 | 1.584(0.752,3.336) | 1.139(0.537,2.418) | 1.072(0.523,2.200) | 2.945(1.467,5.911) | 2.249(1.082,4.673) | 0.597(0.299,1.193) | 0.973(0.477,1.981) |
| 15< AHI≤30 | 1.449(0.637,3.292) | 2.071(0.934,4.594) | 2.490(1.149,5.399) | 2.433(1.117,5.301) | 1.656(0.723,3.791) | 0.604(0.283,1.288) | 0.994(0.462,2.141) |
| AHI>30 | 2.511(1.196,5.274) | 2.769(1.299,5.899) | 2.382(1.137,4.990) | 3.641(1.729,7.668) | 2.365(1.089,5.138) | 1.287(0.661,2.507) | 2.675(1.349,5.305) |
| P-value for linear trend | 0.010^*^ | 0.003^*^ | 0.014^*^ | 0.005^*^ | 0.099 | 0.403 | 0.010^*^ |

**Notes:** ORs were adjusted for age, body mass index (BMI), sex and plus Smoking status, Alcohol consumption, mean artierial pressure in model 2 and plus MAI in model 3; P-values for linear trends were determined by examining the median AHI value for each quartile and assessing the overall F test for the median MAI variable. *P<0.05.

**Abbreviations:** AHI, apnea-hyponea index.

**Table S27** Adjusted odds ratios for abnormal glucose and lipid metabolism according to CT90 categories in models 1, 2 and 3 **inFemale subjects**

|  | Hyperglycemia | Hyperinsulinemia | HOMA-IR≥2.5 | Hyper-total cholesterolemia | Hyper-LDL cholesterolemia | Hypo-HDL cholesterolemia | Hyper- triglyceridemia |
| --- | --- | --- | --- | --- | --- | --- | --- |
| Adjusted OR(95% CI) in model 1 | | | | | | | |
| CT90≤0.0188 | 1 | 1 | 1 | 1 | 1 | 1 | 1 |
| 0.0188< CT90≤1.6 | 1.140(0.617,2.107) | 1.382(0.761,2.507) | 1.453(0.824,2.564) | 2.046(1.143,3.662) | 1.777(0.932,3.389) | 1.048(0.622,1.767) | 2.042(1.130,3.691) |
| CT90>1.6 | 1.116(0.563,2.213) | 1.592(0.793,3.194) | 1.662(0.850,3.249) | 1.858(0.969,3.561) | 2.270(1.119,4.606) | 1.141(0.612,2.126) | 2.296(1.195,4.411) |
| P-value for linear trend | 0.019^*^ | 0.347 | 0.605 | 0.791 | 0.986 | 0.789 | 0.164 |
| Adjusted OR(95% CI) in model 2 | | | | | | | |
| CT90≤0.0188 | 1 | 1 | 1 | 1 | 1 | 1 | 1 |
| 0.0188< CT90≤1.6 | 1.013(0.532,1.930) | 1.281(0.684,2.399) | 1.374(0.759,2.487) | 2.001(1.095,3.658) | 1.619(0.838,3.130) | 0.987(0.577,1.687) | 2.183(1.183,4.029) |
| CT90>1.6 | 0.994(0.481,2.054) | 2.012(0.959,4.221) | 2.054(1.003,4.203) | 1.828(0.913,3.659) | 1.891(0.900,3.972) | 1.148(0.599,2.201) | 2.625(1.312,5.251) |
| P-value for linear trend | 0.027^*^ | 0.102 | 0.261 | 0.936 | 0.602 | 0.653 | 0.098 |
| Adjusted OR(95% CI) in model 3 | | | | | | | |
| CT90≤0.0188 | 1 | 1 | 1 | 1 | 1 | 1 | 1 |
| 0.0188< CT90≤1.6 | 1.024(0.536,1.957) | 1.277(0.679,2.403) | 1.373(0.755,2.497) | 2.210(1.199,4.072) | 1.744(0.897,3.390) | 0.986(0.575,1.690) | 2.125(1.149,3.931) |
| CT90>1.6 | 1.018(0.486,2.136) | 1.997(0.934,4.273) | 2.051(0.983,4.281) | 2.327(1.135,4.771) | 2.329(1.084,5.004) | 1.146(0.591,2.222) | 2.435(1.203,4.927) |
| P-value for linear trend | 0.018^*^ | 0.121 | 0.302 | 0.458 | 0.910 | 0.662 | 0.190 |

**Notes:** ORs were adjusted for age, body mass index (BMI), sex and plus Smoking status, Alcohol consumption, mean artierial pressure in model 2 and plus MAI in model 3; P-values for linear trends were determined by examining the median CT90 value for each quartile and assessing the overall F test for the median MAI variable. *P<0.05.

**Abbreviations:** CT90, percentage of total sleep time with SpO_2_<90%.

**Table S28** The result of Spearman correlation

|  |  | Age | BMI | Sex | Smoking | Alcohol | MAP | MAI | SASHB |
| --- | --- | --- | --- | --- | --- | --- | --- | --- | --- |
| Age | Spearman Correlation | 1 | 0.005 | .178** | 0.032 | -0.015 | 0.034 | .060** | .177** |
|  | Sig.(2-tailed) | . | 0.824 | 0 | 0.182 | 0.529 | 0.155 | 0.009 | 0 |
|  | N | 1911 | 1854 | 1911 | 1773 | 1773 | 1773 | 1906 | 1911 |
| BMI | Spearman Correlation | 0.005 | 1 | -.171** | 0.033 | .088** | .065** | .170** | .361** |
|  | Sig.(2-tailed) | 0.824 | . | 0 | 0.163 | 0 | 0.006 | 0 | 0 |
|  | N | 1854 | 1899 | 1898 | 1770 | 1770 | 1770 | 1895 | 1899 |
| Sex | Spearman Correlation | .178** | -.171** | 1 | -.090** | -.107** | .079** | -.179** | -.176** |
|  | Sig.(2-tailed) | 0 | 0 | . | 0 | 0 | 0 | 0 | 0 |
|  | N | 1911 | 1898 | 2161 | 1999 | 1999 | 1999 | 1962 | 2161 |
| Smoking | Spearman Correlation | 0.032 | 0.033 | -.090** | 1 | -.139** | 0.018 | -0.036 | .049* |
|  | Sig.(2-tailed) | 0.182 | 0.163 | 0 | . | 0 | 0.43 | 0.127 | 0.03 |
|  | N | 1773 | 1770 | 1999 | 2001 | 2001 | 2001 | 1826 | 2001 |
| Alcohol | Spearman Correlation | -0.015 | .088** | -.107** | -.139** | 1 | 0.018 | .078** | .076** |
|  | Sig.(2-tailed) | 0.529 | 0 | 0 | 0 | . | 0.432 | 0.001 | 0.001 |
|  | N | 1773 | 1770 | 1999 | 2001 | 2001 | 2001 | 1826 | 2001 |
| MAP | Spearman Correlation | 0.034 | .065** | .079** | 0.018 | 0.018 | 1 | -.064** | 0.001 |
|  | Sig.(2-tailed) | 0.155 | 0.006 | 0 | 0.43 | 0.432 | . | 0.006 | 0.948 |
|  | N | 1773 | 1770 | 1999 | 2001 | 2001 | 2001 | 1826 | 2001 |
| MAI | Spearman Correlation | .060** | .170** | -.179** | -0.036 | .078** | -.064** | 1 | .393** |
|  | Sig.(2-tailed) | 0.009 | 0 | 0 | 0.127 | 0.001 | 0.006 | . | 0 |
|  | N | 1906 | 1895 | 1962 | 1826 | 1826 | 1826 | 1963 | 1963 |
| SASHB | Spearman Correlation | .177** | .361** | -.176** | .049* | .076** | 0.001 | .393** | 1 |
|  | Sig.(2-tailed) | 0 | 0 | 0 | 0.03 | 0.001 | 0.948 | 0 | . |
|  | N | 1911 | 1899 | 2161 | 2001 | 2001 | 2001 | 1963 | 2184 |

**. Correlation is significant at the 0.01 level (2-tailed).

**Table S29** Result of Collinearity Diagnostics

| Model |  | Unstandardized Coefficients | | Standardized Coefficients | t | Sig. | Collinearity Statistics | |
| --- | --- | --- | --- | --- | --- | --- | --- | --- |
|  |  | B | Std.Error | Beta |  |  | Tolerance | VIF |
| 1 | (Constant) | 2.441 | 0.272 |  | 8.98 | 0 |  |  |
|  | Age | 0.017 | 0.002 | 0.186 | 7.855 | 0 | 0.933 | 1.071 |
|  | BMI | 0.076 | 0.008 | 0.242 | 9.946 | 0 | 0.892 | 1.122 |
|  | Sex | -0.002 | 0.065 | -0.001 | -0.029 | 0.977 | 0.905 | 1.104 |
|  | Smoking | -0.22 | 0.555 | -0.009 | -0.397 | 0.691 | 0.987 | 1.013 |
|  | Alcohol | 0.042 | 0.178 | 0.006 | 0.238 | 0.812 | 0.979 | 1.021 |
|  | MAP | 0.072 | 0.064 | 0.026 | 1.116 | 0.265 | 0.975 | 1.025 |
|  | MAI | 0.003 | 0.002 | 0.04 | 1.654 | 0.098 | 0.883 | 1.132 |
|  | SASHB | 0 | 0 | 0.046 | 1.797 | 0.073 | 0.805 | 1.243 |

1. Dependent Variable: FBG

**Table S30** Result of Collinearity Diagnostics.

| Model | Dimension | Eigenvalue | Condition Index | | Variance Proportions | | | | | | | |
| --- | --- | --- | --- | --- | --- | --- | --- | --- | --- | --- | --- | --- |
|  |  |  |  |  | Age | BMI | Sex | Smoking | Alcohol | MAP | MAI | SASHB |
| 1 | 1 | 6.178 | 1 | 0 | 0 | 0 | 0.01 | 0 | 0 | 0 | 0.01 | 0.01 |
|  | 2 | 1.002 | 2.483 | 0 | 0 | 0 | 0 | 0.97 | 0 | 0 | 0 | 0 |
|  | 3 | 0.826 | 2.735 | 0 | 0 | 0 | 0.62 | 0 | 0 | 0 | 0.03 | 0.09 |
|  | 4 | 0.428 | 3.8 | 0 | 0 | 0 | 0.31 | 0.01 | 0 | 0.06 | 0.07 | 0.47 |
|  | 5 | 0.301 | 4.528 | 0 | 0 | 0 | 0 | 0 | 0 | 0.04 | 0.8 | 0.36 |
|  | 6 | 0.18 | 5.862 | 0 | 0.04 | 0 | 0 | 0 | 0.01 | 0.86 | 0.1 | 0.01 |
|  | 7 | 0.059 | 10.23 | 0.01 | 0.89 | 0.03 | 0.05 | 0.01 | 0.05 | 0.02 | 0 | 0 |
|  | 8 | 0.02 | 17.716 | 0 | 0 | 0.42 | 0 | 0 | 0.62 | 0.01 | 0 | 0.01 |
|  | 9 | 0.007 | 30.124 | 0.98 | 0.07 | 0.54 | 0.02 | 0 | 0.33 | 0 | 0 | 0.05 |

1. Dependent Variable: FBG

**Description of QC criteria for SpO_2_ trend:**

The calculation of the SASHB parameter was based on the sum of the areas included in the SpO_2_ trend map at a set baseline within the window of respiratory events.The quality control criterion for the length of SpO_2_ recordings was defined as an effective recording length of $\geq$4 h. For the quality control criterion of artefacts, the quality of SpO_2_ signals was not affected by the interference of smaller movement artefacts and sweat artefacts, so we deleted the movement artefacts and shedding of artefacts due to larger body movements. Therefore, we removed motion artefacts caused by large body movements and abnormal SpO_2_ values caused by shedding, and the effective recording duration should be$\geq$4 hours.

***Original Codes of calculating SASHB:***

clear all

clc

[hdr, record] = edfread('A000A.edf');

[m,n]=size(record);

hour=n/100/3600;

data=record(15,1:n);

data1=round(data);

len=length(data1);

[LSaO2,AveSaO2,CT90,oi,ODBeginXarray,ODBeginYarray,ODXarray,ODYarray,ODupBeginXarray,ODupBeginYarray,ODupEndXarray,ODupEndYarray,ODupTime] = FindSpO2Value(data1);

for i=1:len

if data1(i)==0

data1(i)=AveSaO2;

end

if data1(i)>100

data1(i)=100;

end

end

figure(1);

subplot(211);

ylim([70 100]);

plot(data1);

subplot(212);

ylim([70 100]);

plot(data1);

hold on

plot(ODXarray,ODYarray,'r<');

hold on

plot(ODBeginXarray,ODBeginYarray,'r>');

hold on

plot(ODupEndXarray,ODupEndYarray,'b<');

hold on

plot(ODupBeginXarray,ODupBeginYarray,'r>');

hold on

n1=length(ODBeginXarray);

n2=length(ODupEndXarray);

md=[];

if n1==n2

nn=n1;

nn=n2;

end

if n1~=n2

n1=min(n1,n2);

n2=min(n1,n2);

nn=n1;

nn=n2;

end

disp(nn);

for k=1:nn

diff(k)=ODupEndXarray(k)-ODBeginXarray(k);

end

disp(diff);

sumdiff=sum(diff);

for mm=1:nn

for t=1:diff(mm)

chay(t)=data1(ODBeginXarray(mm))-data1(ODBeginXarray(mm)+t);

chay1(t)=chay(t)/6000;

end

disp(chay); quy(mm)=sum(chay1);

sashb(mm)=quy(mm);

end

disp(sashb);

sumquy=sum(quy1);

sumsashb=sum(sashb);

sashb=sumsashb/hour;

sashb1=roundn(sashb,-2);

sumsashb1=sum(sashb1);

function [ifindNRPeakraw, ifindNRPeakcolumn, ifindNRValleyraw, ifindNRValleycolumn, NRPeak,NRValley] = Find_Resp_Peak_Valley_Point3(NRLength,NewN)

iNRPeakraw = [];

iNRPeakcolumn = [];

iNRValleyraw = [];

iNRValleycolumn = [];

ifindNRPeakraw = [];

ifindNRPeakcolumn = [];

ifindNRValleyraw = [];

ifindNRValleycolumn = [];

fNRmaxdetect = 0;

fNRmindetect = 0;

fmax1 = 0;

fNRPercent = 0;

NRmax = 0;

NRespmin = 0;

NRj = 1;

NRt = 1;

NRk = 1;

NRg = 1;

i = 1;

fmin1 = 0;

NRmaxi = 1;

NRmini = 1;

iNRPeakpp = 0;

NRmin = 180;

NRmaxg = 1;

NRming = 1;

Aveflag = 1;

j = 1;

Max1 = 1;

Min1 = 1;

FRPflag = 0;

FRVflag = 0;

for i = 1:NRLength

NRespflow = NewN(i);

if (NRespflow > 180)

if (fNRmaxdetect == 0)

if (NRespflow > NRmax)

NRmax = NRespflow;

else

iNRPeakraw(NRj,1) = i-1;

iNRPeakvv = i-1;

iNRPeakcolumn(NRj,1) = NRmax;

iNRPeakpp = NRmax;

NRj = NRj + 1;

fNRmaxdetect = 1;

end

end

if (fNRmaxdetect == 1)

fNRmaxpercent = ((NRmax-180) / (NRespflow-180));

NRmaxi = NRmaxi + 1;

if (fNRmaxpercent > 3)

if (iNRPeakvv > NRmaxi)

fmax1 = (NRmax - NewN(iNRPeakvv - NRmaxi));

NRmaxg = (NewN(i) - NewN(i - 10));

end

if ((fmax1 > 0) && (NRmaxg > 0))

ifindNRPeakcolumn(NRt,1) = NRmax;

ifindNRPeakraw(NRt,1) = iNRPeakvv;

NRt = NRt + 1;

fNRmaxdetect = 0;

NRmax =179;

NRmaxi = 1;

end

end

if (fNRmaxpercent < 1)

fNRmaxdetect = 0;

NRmax = 179;

end

end

end

if (NRespflow < 1000)

if (fNRmindetect == 0)

if (NRespflow < NRmin)

NRmin = NRespflow;

else

iNRValleyraw(NRk,1) = NRmin;

iValleypp = NRmin;

iNRValleycolumn(NRk,1) = i - 1;

iValleyvv = i - 1;

fNRmindetect = 1;

NRk = NRk + 1;

end

end

if (fNRmindetect == 1)

fNRminpercent = ((NRmin - 1000) / (NRespflow - 1000));

NRmini = NRmini + 1;

if (fNRminpercent > 2)

if(iValleyvv > NRmini)

fmin1 = (NRmin - NewN(iValleyvv - NRmini));

NRming = (NewN(i) - NewN(i - 10));

end

if ((fmin1 < 0) && (NRming < 0))

ifindNRValleycolumn(NRg,1) = NRmin;

ifindNRValleyraw (NRg,1) = iValleyvv;

NRg = NRg + 1;

NRmin = 1001;

fNRmindetect = 0;

NRmini = 1;

end

end

if (fNRminpercent < 1)

fNRmindetect = 0;

NRmin = 1001;

end

end

end

end

NRPeak = NRt - 1;

NRValley = NRg - 1;

end
